# Intestinal α-Defensins Play a Minor Role in Modulating the Small Intestinal Microbiota Composition as Compared to Diet

**DOI:** 10.1128/spectrum.00567-23

**Published:** 2023-04-11

**Authors:** Fabiola Puértolas-Balint, Bjoern O. Schroeder

**Affiliations:** a Department of Molecular Biology, Umeå University, Umeå, Sweden; b The Laboratory for Molecular Infection Medicine Sweden (MIMS), Umeå University, Umeå, Sweden; c Umeå Center for Microbial Research (UCMR), Umeå University, Umeå, Sweden; Lerner Research Institute

**Keywords:** defensins, gut microbiota, metabolic disease, mucosal barrier, Western diet, antimicrobial peptides

## Abstract

The intestinal microbiota is at the interface between the host and its environment and thus under constant exposure to host-derived and external modulators. While diet is considered to be an important external factor modulating microbiota composition, intestinal defensins, one of the major classes of antimicrobial peptides, have been described as key host effectors that shape the gut microbial community. However, since dietary compounds can affect defensin expression, thereby indirectly modulating the intestinal microbiota, their individual contribution to shaping gut microbiota composition remains to be defined. To disentangle the complex interaction among diet, defensins, and small-intestinal microbiota, we fed wild-type (WT) mice and mice lacking functionally active α-defensins (*Mmp7^–/−^* mice) either a control diet or a Western-style diet (WSD) that is rich in saturated fat and simple carbohydrates but low in dietary fiber. 16S rDNA sequencing and robust statistical analyses identified that bacterial composition was strongly affected by diet while defensins had only a minor impact. These findings were independent of sample location, with consistent results between the lumen and mucosa of the jejunum and ileum, in both mouse genotypes. However, distinct microbial taxa were also modulated by α-defensins, which was supported by differential antimicrobial activity of ileal protein extracts. As the combination of WSD and defensin deficiency exacerbated glucose metabolism, we conclude that defensins only have a fine-tuning role in shaping the small-intestinal bacterial composition and might instead be important in protecting the host against the development of diet-induced metabolic dysfunction.

**IMPORTANCE** Alterations in the gut microbial community composition are associated with many diseases, and therefore identifying factors that shape the microbial community under homeostatic and diseased conditions may contribute to the development of strategies to correct a dysbiotic microbiota. Here, we demonstrate that a Western-style diet, as an extrinsic parameter, had a stronger impact on shaping the small intestinal bacterial composition than intestinal defensins, as an intrinsic parameter. While defensins have been previously shown to modulate bacterial composition in young mice, our study supplements these findings by showing that defensins may be less important in adult mice that harbor a mature microbial community. Nevertheless, we observed that defensins did affect the abundance of distinct bacterial taxa in adult mice and protected the host from aggravated diet-induced glucose impairments. Consequently, our study uncovers a new angle on the role of intestinal defensins in the development of metabolic diseases in adult mice.

## INTRODUCTION

The gut microbiota comprises a diverse community of microorganisms that affect metabolism, brain function, immunity, nutrition, and development of the host (1 to 3). While many of these functions are beneficial, the enormous number of microbial organisms that are in close proximity to the host epithelium poses a permanent risk of intestinal infection and inflammation. Consequently, the host has evolved site-specific defense mechanisms to control and shape the microbial community in the gut. Such mechanisms include the presence of a thick mucus layer, the secretion of immunoglobulin A (sIgA), and the production of antimicrobial peptides (AMPs) (4 to 7).

Intestinal AMPs are small cationic peptides that prevent bacteria from reaching the host epithelium by permeabilizing the bacterial membrane or by limiting their mobility through aggregation (8, 9). The most abundant AMPs in the mouse intestine are α-defensins (also known as cryptdins), produced by Paneth cells (6, 10). Mice additionally produce a unique family of AMPs called cryptdin-related sequences (CRS) peptides, which share sequence similarity with defensins and form homo- and heterodimers that differ in their antimicrobial activities (11). To become active, defensins and CRS peptides require proteolytic processing by the matrix metalloproteinase 7 (Mmp7, also called matrilysin), prior to their release into the small intestinal crypts (12, 13). While proteolytic activation by Mmp7 is a posttranscriptional control of defensin activity, defensins are also regulated on the transcriptional level. As such, by using germ-free or antibiotic-treated mouse models, it has been shown that the presence of gut bacteria is required for full induction of AMP expression (14, 15), which suggests that commensal gut bacteria can induce AMP expression.

AMPs protect the host against lethal infection by intestinal pathogens such as Salmonella typhimurium and Shigella flexneri (13, 16) but are generally thought to lack antimicrobial activity toward commensal bacteria (17, 18). However, we and others have shown that environmental conditions affect the activity spectrum of defensins and that, under conditions relevant to the intestine, considerable antimicrobial activity against commensal bacteria is detected *in vitro* (17, 19–21). These findings have been corroborated by two complementary mouse models being either deficient in functional α-defensins (*Mmp7^−/−^* deficient mice) or transgenic for the expression of human α-defensin 5 (HD5), in which the composition of the small intestinal bacterial community was modulated by defensins (22). However, when using the same *Mmp7^−/−^* mouse model, other studies did not observe a significant effect of defensins on the small intestinal bacterial composition (23, 24), thus challenging the relevance of defensins in shaping the intestinal microbiota composition.

Besides AMPs, dietary fiber is a critical modulator of intestinal microbiota composition (25). However, modern dietary habits are often characterized by diets low in fibers and rich in sugars and fat, and intake of this “Western-style diet” (WSD) has been linked to inflammatory bowel diseases (IBD), including ulcerative colitis and Crohn’s disease (26). As defensin expression is reduced in patients with ileal Crohn’s disease and increased in patients with ulcerative colitis (27, 28), the combination of altered AMP expression and diet may shape an environment that promotes a dysbiotic microbiota, thereby contributing to disease progression.

WSD is also a major contributor to the development of metabolic disorders, including diabetes and obesity (29). Interestingly, obese individuals have reduced protein levels of HD5 and intestinal lysozyme, and lysozyme levels correlated negatively with body mass index (BMI) (30). Moreover, as several mouse studies have observed altered AMP expression and shifted microbiota composition upon WSD consumption (31, 32), it is possible that diet also shapes the microbial community indirectly through modulating AMP expression. However, the specific contribution of AMPs and diet to the microbiota composition remains so far unclear.

To disentangle the individual roles of diet and intestinal defensins in shaping the microbiota composition in mice, we here combined the usage of the defensin-deficient *Mmp7^−/−^* mouse model, a WSD intervention, and robust statistical analyses to analyze the small intestinal microbiota composition. We specifically investigated the bacterial composition in the small intestine where α-defensin production is highest, and we further evaluated whether the combination of WSD-feeding and defensin deficiency exacerbates host metabolism in the mice.

## RESULTS

### Diet has a stronger effect on small intestinal microbiota composition than defensins.

**FIG 1:**
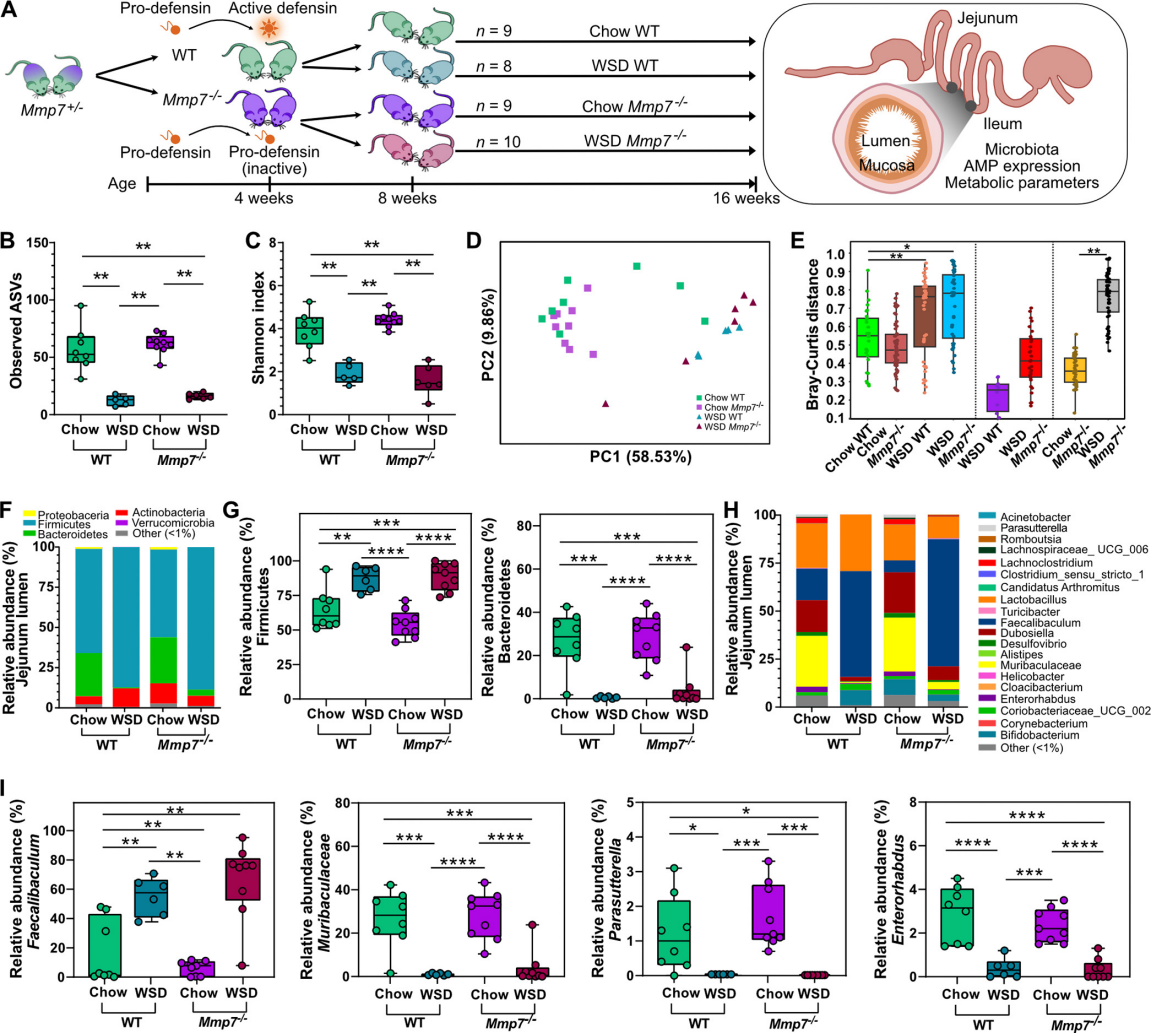
Diet is a stronger modulator of luminal microbiota composition in the jejunum than defensins. (A) Schematic experimental setup: littermate-controlled WT or *Mmp7^−/−^* mice were fed a chow or Western-style diet (WSD) for 8 weeks. Alpha diversity according to (B) observed ASVs and (C) Shannon index and beta diversity according to (D) Bray-Curtis dissimilarity matrix and (E) Bray-Curtis distance in the jejunum lumen. (F) Average phylum relative abundance and (G) differentially abundant phyla, (H) average genera relative abundance and (I) differentially abundant genera in the jejunum lumen. Phyla or genera with less than 1% relative abundance are represented as “other (<1%).” Statistical tests calculated with the Kruskal-Wallis Pairwise test for alpha-diversity analyses, PERMANOVA for beta-diversity analyses and pairwise PERMANOVA for Bray-Curtis distance (presented as p_adj_), and two-way ANOVA with Tukey’s multiple-comparison test of phyla and genera. *, *P* ≤ 0.05; **, *P* ≤ 0.01; ***, *P* ≤ 0.001; ****, < 0.0001.

To disentangle the complex interdependence among defensins, diet, and microbiota (33), wild-type (WT) mice and mice deficient in Mmp7 (Fig. S1 in the supplemental material) were fed a WSD or a control chow diet (Fig. 1A), and the bacterial composition of the jejunum and ileum in the lumen and at the mucosa was investigated.

In the jejunal lumen, we observed significantly larger differences in alpha-diversity measures between the two diets, including number of observed amplicon sequencing variants (ASVs; *P* = 0.003, Fig. 1B) and Shannon diversity index (*P* = 0.005, Fig. 1C), while the presence or absence of active defensins did not alter these measures of alpha diversity during chow (*P* = 0.268; *P* = 0.210) or WSD feeding (*P* = 0.118; *P* = 0.465). The dominating effect of diet was also confirmed by beta-diversity analyses in WT and *Mmp7^−/−^* mice using a Bray-Curtis dissimilarity matrix (*P*_adj_ = 0.004; *P*_adj_ = 0.003; Fig. 1D, E) and unweighted (*P*_adj_ = 0.002; *P*_adj_ = 0.002; Fig. S2A) or weighted (*P*_adj_ = 0.002; *P*_adj_ = 0.002; Fig. S2B) UniFrac distance matrices, demonstrating significantly higher similarity between mouse groups that were fed the same diet, independent of α-defensins. However, the Bray-Curtis dissimilarity matrix also identified a weak yet statistically significant role of the defensins on the jejunal bacterial composition under chow diet feeding (*P* = 0.049, Fig. 1E), but this difference was lost after correcting for multiple comparisons (*P*_adj_ = 0.058).

When comparing the relative abundance of microbial taxa in the jejunum, WSD feeding resulted in decreased abundance of Bacteroidetes and increased abundance of Firmicutes at the phylum level, independent of genotype (Fig. 1F, G). At the genus level, *Faecalibaculum* was significantly enriched in WT and defensin-deficient mice upon WSD feeding, while the abundance of *Parasutterella*, *Enterorhabdus*, and *Muribaculaceae* was decreased in both genotypes (Fig. 1H, I). Hence, in line with the diversity analyses, using diet and genotype as two independent variables, the microbial abundance in the jejunal lumen was strongly modulated by diet but not by defensins (Table S1). One exception, however, was the abundance of Actinobacteria and its genus *Bifidobacterium*, for which the diet-genotype interaction significantly affected the abundance (*P* = 0.011; *P* = 0.017) (Table S1).

**FIG 2:**
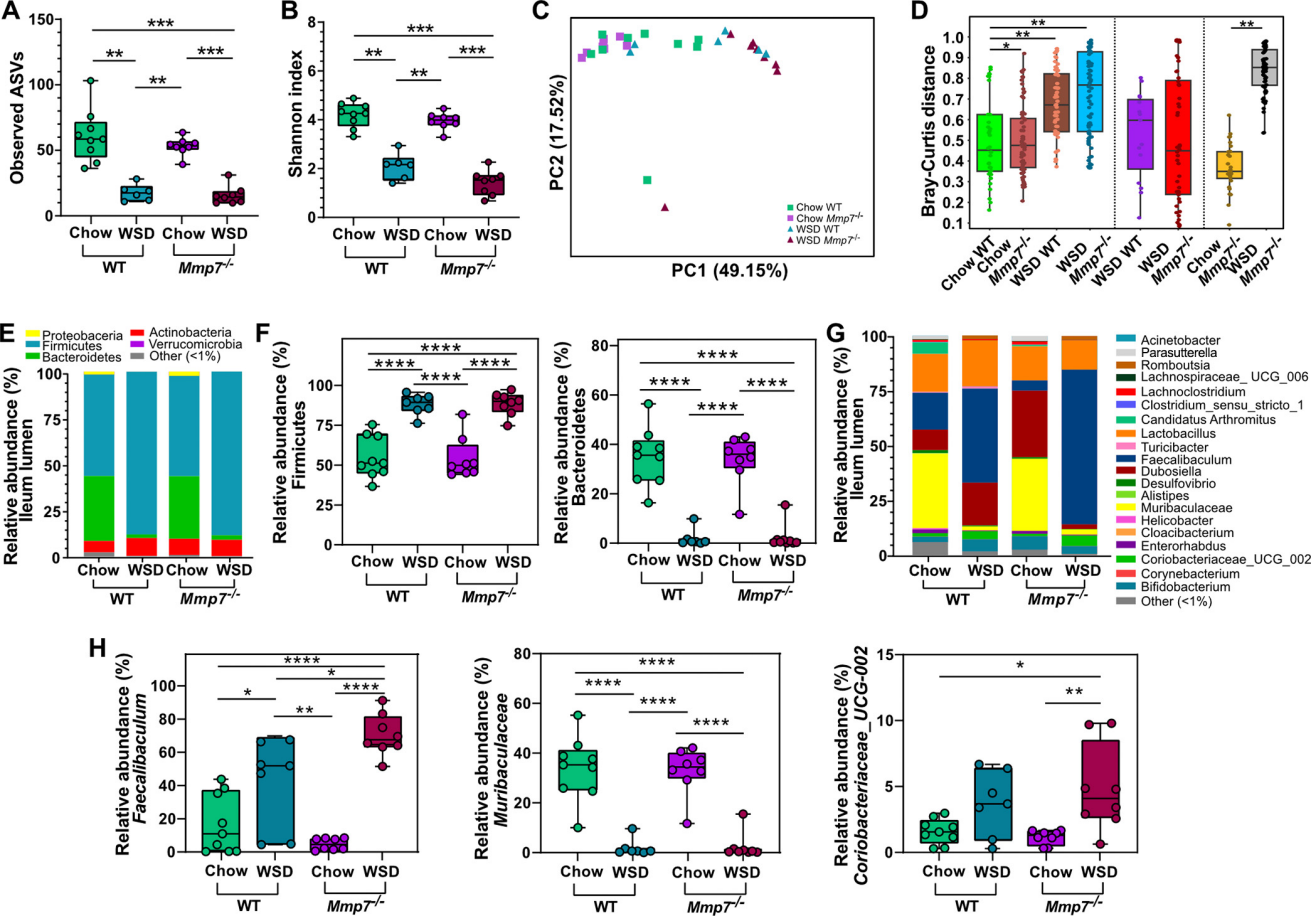
Diet is a stronger modulator of luminal microbiota composition than defensins in the ileum. Alpha diversity according to (A) observed ASVs and (B) Shannon index, and beta diversity according to (C) Bray-Curtis dissimilarity matrix and (D) Bray-Curtis distance at the ileum lumen. (E) Average phylum relative abundance and (F) differentially abundant phyla, (G) average genera relative abundance and (H) differentially abundant genera at the ileum lumen. Phyla or genera with less than 1% relative abundance are represented as “other (<1%).” Statistical tests calculated with Kruskal-Wallis Pairwise test for alpha-diversity analyses, PERMANOVA for beta-diversity analyses and pairwise PERMANOVA for Bray-Curtis distance (presented as p_adj_), and two-way ANOVA with Tukey’s multiple-comparison test of phyla and genera. *, *P* ≤ 0.05; **, *P* ≤ 0.01; ***, *P* ≤ 0.001; ****, *P* < 0.0001.

Similarly, in the ileal lumen, the number of observed ASVs (Fig. 2A) and the Shannon index (Fig. 2B) were also strongly affected by diet in WT (*P* = 0.001; *P*= 0.001) and *Mmp7^−/−^* mice (*P* = 0.001; *P*= 0.0007), but not by defensins, neither on a chow diet (*P* = 0.267; *P* = 0.148) nor on WSD feeding (*P* = 0.330; *P* = 0.093), thus confirming the lack of influence of defensins on alpha diversity in the small intestine. Furthermore, diet led to significantly distinct microbial clusters after three different beta-diversity analyses in both WT and *Mmp7^−/−^* mice (Bray-Curtis: *P*_adj_ = 0.001; *P*_adj_ = 0.001, Fig. 2C, D; unweighted UniFrac: *P*_adj_ = 0.003; *P*_adj_ = 0.002, Fig. S2C; weighted UniFrac: *P*_adj_ = 0.001; *P*_adj_ = 0.001, Fig. S2D). However, corroborating the jejunal analysis, defensin deficiency significantly shifted the bacterial composition in mice feeding on a chow diet when using a Bray-Curtis dissimilarity matrix (*P*_adj_= 0.012, Fig. 2C, D) but not when using unweighted or weighted UniFrac distance matrices (Fig. S2C, D).

In accordance with the results for the jejunum, taxonomic analysis in the ileal lumen identified similar changes under WSD feeding on either genotype, with an increased abundance of Firmicutes (Fig. 2E, F) and *Faecalibaculum* (Fig. 2G, H), while the abundance of Bacteroidetes and *Muribaculaceae* decreased. The absence of active defensins led to a higher abundance of *Coriobacteriaceae_UCG-002* only in WSD-fed mice (Fig. 2H), suggesting that a combination of diet and defensins regulated the abundance of this taxon. Again, the strong effect of diet on the bacterial composition in the ileum was confirmed by comparing the effect of diet and genotype by usage of a two-way ANOVA analysis (Table S1). Taken together, diet has thus a strong effect on the microbial diversity and composition in the small intestinal lumen, whereas α-defensins only had a minor role.

**FIG 3:**
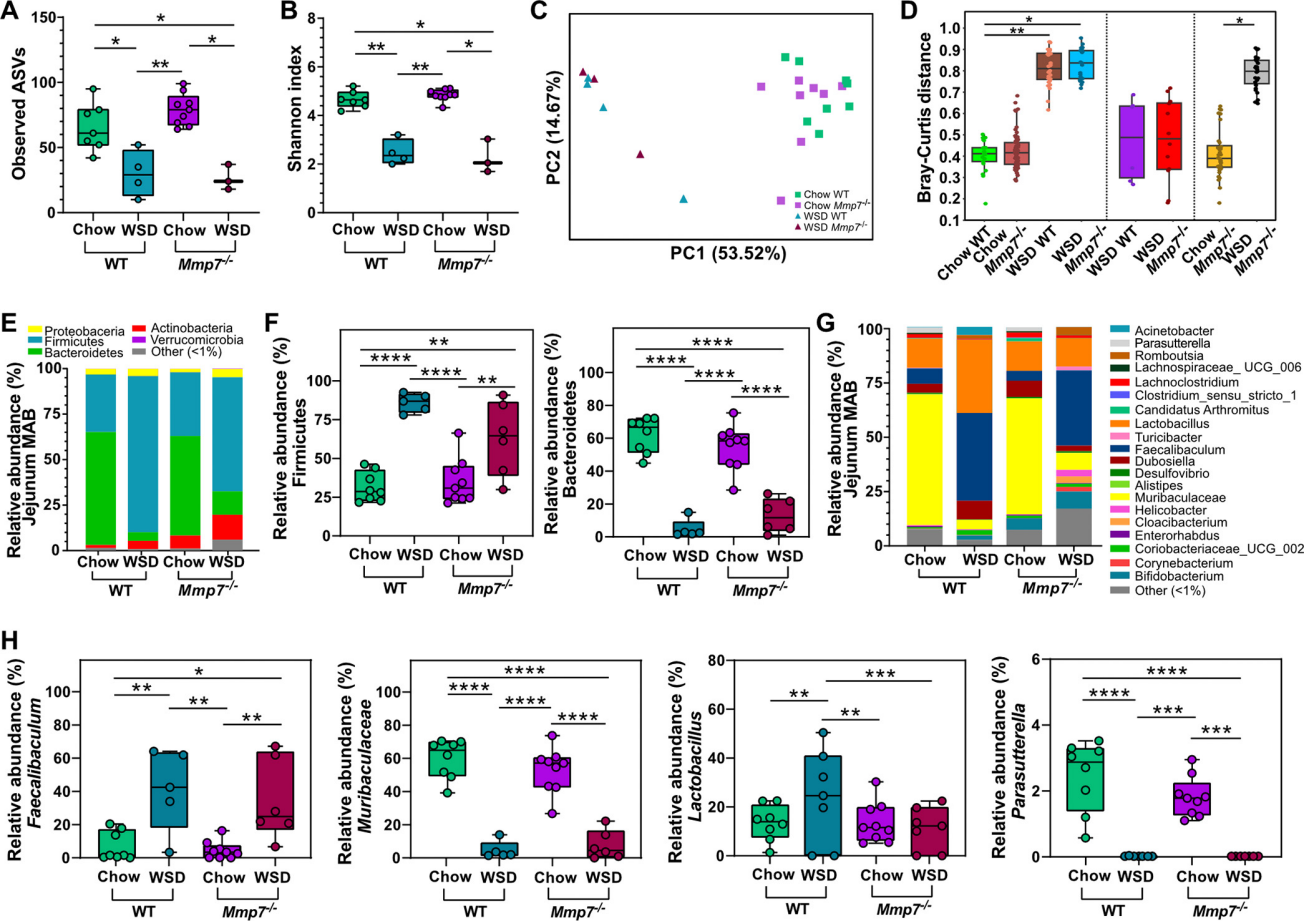
Diet is a stronger modulator of mucosal microbiota composition in the jejunum than defensins. Alpha diversity according to (A) observed ASVs and (B) Shannon index, and beta diversity according to (C) Bray-Curtis dissimilarity matrix and (D) Bray-Curtis distance between chow- or WSD-fed WT and *Mmp7^−/−^* mice observed for the mucosa-associated bacteria (MAB) in the jejunum. (E) Average phylum relative abundance and (F) differentially abundant phyla, (G) average genera relative abundance and (H) differentially abundant genera at the jejunum MAB. Phyla or genera with less than 1% relative abundance are represented as “other (<1%).” Statistical tests calculated with Kruskal-Wallis Pairwise test for alpha-diversity analyses, PERMANOVA for the beta-diversity analyses and pairwise PERMANOVA for Bray-Curtis distance (presented as p_adj_), and two-way ANOVA with Tukey’s multiple-comparison test of phyla and genera. *, *P* ≤ 0.05; **, *P* ≤ 0.01; ***, *P* ≤ 0.001; ****, *P* < 0.0001; ****, *P* < 0.0001.

The closest interaction between the intestinal microbiota and AMPs occurs directly at the mucosal surface. We therefore tested whether defensins have a stronger modulatory role on the mucosa-associated bacteria compared to the bacteria in the lumen. However, similar to the alpha diversity in the jejunal lumen, only diet affected the number of observed ASVs (*P* = 0.017, *P* = 0.012, Fig. 3A) and reduced the Shannon diversity index (*P* = 0.008, *P* = 0.012, Fig. 3B) at the jejunal mucosa of WT and *Mmp7^−/−^* mice, while defensin deficiency did not lead to significant changes. Likewise, diet dominated microbial cluster formation according to the Bray-Curtis dissimilarity matrix (*P*_adj_ = 0.003; *P*_adj_ = 0.012, Fig. 2C, D) and unweighted (*P*_adj_ = 0.010; *P*_adj_ = 0.010, Fig. S2E) or weighted (*P*_adj_ = 0.006; *P*_adj_ = 0.006, Fig. S2F) UniFrac distance matrices, whereas the presence or absence of active defensins did not have any substantial effect. In accordance with the microbial changes in the jejunal lumen (Fig. 1F, G), the taxonomic differences at the jejunal mucosa were characterized by increased abundance of Firmicutes and decreased abundance of Bacteroidetes in mice fed a WSD, independent of the genotype (Fig. 3E, F). Furthermore, the WSD consumption increased the abundance of *Lactobacillus* in the WT and *Faecalibaculum* in both genotypes and resulted in lower abundance of *Muribaculaceae* and *Parasutterella*, also in both genotypes (Fig. 3G, H). However, while diet had the most substantial effect on microbial abundances at the jejunum mucosa (Table S2), the genotype significantly affected Actinobacteria, *Bifidobacterium*, and *Gastranarophilales* as shown by two-way ANOVA variable effect comparison, suggesting a discrete impact of defensins at this site.

**FIG 4:**
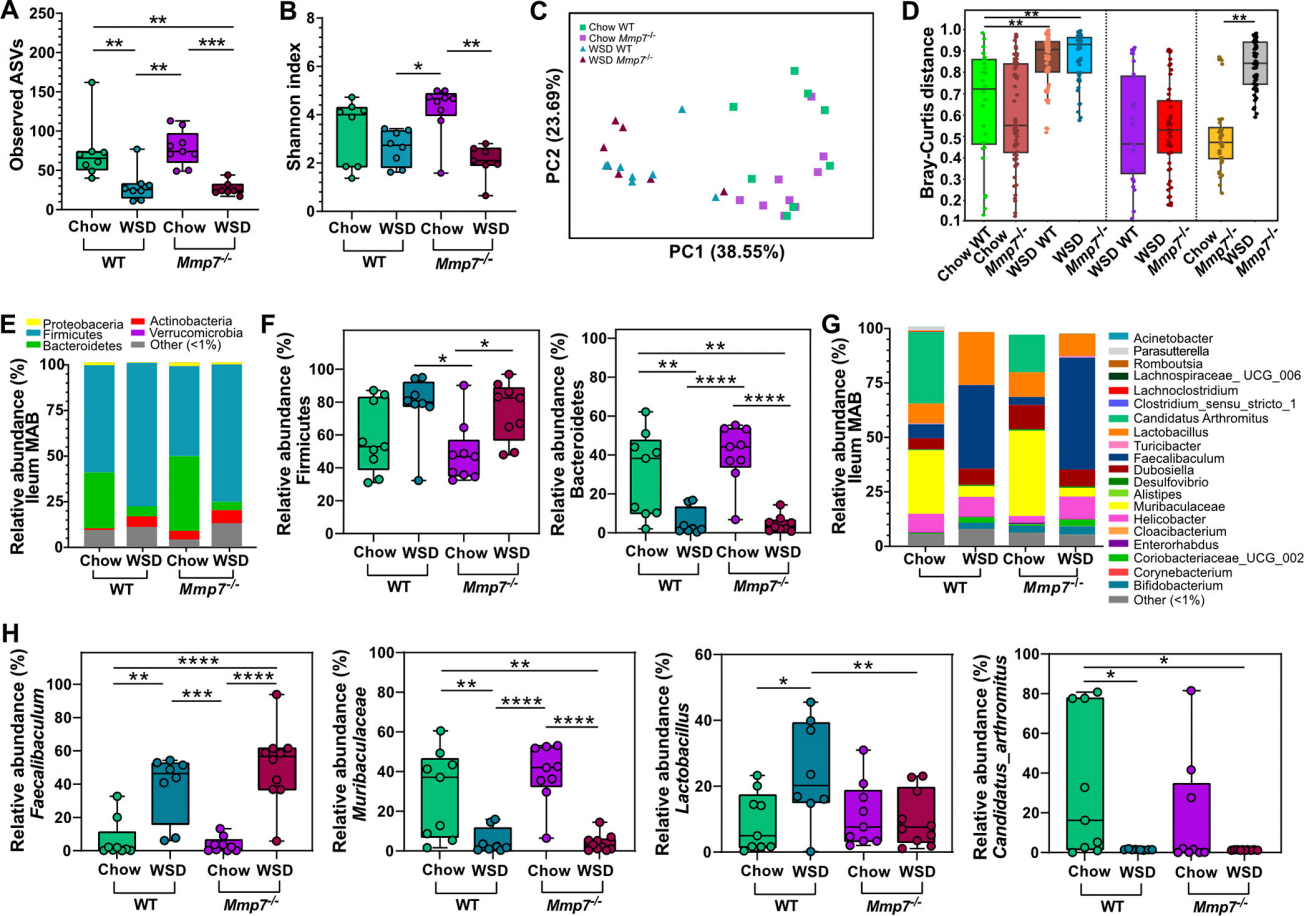
Diet is a stronger modulator of mucosal microbiota composition in the ileum than defensins. Alpha diversity according to (A) observed ASVs and (B) Shannon index and beta diversity according to (C) Bray-Curtis dissimilarity matrix and (D) Bray-Curtis distance between chow- or WSD-fed WT and *Mmp7^−/−^* mice observed for the mucosa-associated bacteria (MAB) in the ileum. (E) Average phylum relative abundance and (F) differentially abundant phyla, (G) average genera relative abundance and (H) differentially abundant genera at the ileum MAB. Phyla or genera with less than 1% relative abundance are represented as “other (<1%).” Statistical tests calculated with Kruskal-Wallis Pairwise test for alpha-diversity analyses, PERMANOVA for beta-diversity analyses and pairwise PERMANOVA for Bray-Curtis distance (presented as p_adj_), and two-way ANOVA with Tukey’s multiple-comparison test of phyla and genera. *, *P* ≤ 0.05; **, *P* ≤ 0.01; ***, *P* ≤ 0.001; ****, *P* < 0.0001; ****, *P* < 0.0001.

In contrast to the previous locations, diet treatment only affected the number of observed ASVs (*P* = 0.008) but not the Shannon diversity index (*P* = 0.207) (Fig. 4A, B) at the ileal mucosa in WT mice. Since defensin deficiency also failed to cause significant changes in alpha diversity under both diet treatments, this suggests that microbial alpha diversity at the ileal mucosa may be more resilient and less affected by host or environmental modulators. However, diet significantly affected beta diversity at the ileal mucosa in WT and *Mmp7^−/−^* mice (Bray-Curtis: *P*_adj_ = 0.001; *P*_adj_ = 0.001, Fig. 4C, D; unweighted UniFrac: *P*_adj_ = 0.003; *P*_adj_ = 0.002, Fig. S2G; weighted UniFrac: *P*_adj_ = 0.001; *P*_adj_ = 0.001, Fig. S2H), while defensin deficiency failed to cause any significant clustering, thus corroborating our previous conclusion that diet is a stronger modulator of small-intestinal microbial diversity than defensins.

Diet also had a significant impact on the phyla at the ileum mucosa (Fig. 4E, F) with similar changes as observed at the jejunum mucosa. At the genus level, lower abundance of *Faecalibaculum* and *Lactobacillus* was detected in both genotypes of chow-fed mice compared to the WSD (Fig. 4G, H). Moreover, the genus *Muribaculaceae* was enriched in chow-fed WT and *Mmp7^−/−^* mice compared to WSD-fed mice, as was *Candidatus arthromitus*, a segmented filamentous bacterium (SFB) known as a potent mucosal immune modulator (34, 35), which was absent in mice fed the WSD (Fig. 4H). In agreement with observations made for the jejunum and ileal lumen, diet also substantially impacted the relative abundance of selected taxa at the ileum mucosa (Table S2). However, using a variable effect comparison identified that the interaction between diet and genotype influenced the abundance of *Lachnosclostridium*, *Marvinbryantia*, [*Eubacterium*]*_brachy_group*, and *Lactobacillus* (Table S2).

Consequently, our analysis shows that WSD has a substantial impact on small intestinal microbial diversity and structure, while the effect of defensins is rather modest. However, some genera were differentially abundant between genotypes fed the same diet, suggesting that defensin deficiency had a discrete impact on individual taxa in the jejunum and the ileum.

### Defensins modulate distinct bacterial taxa at different locations.

**FIG 5:**
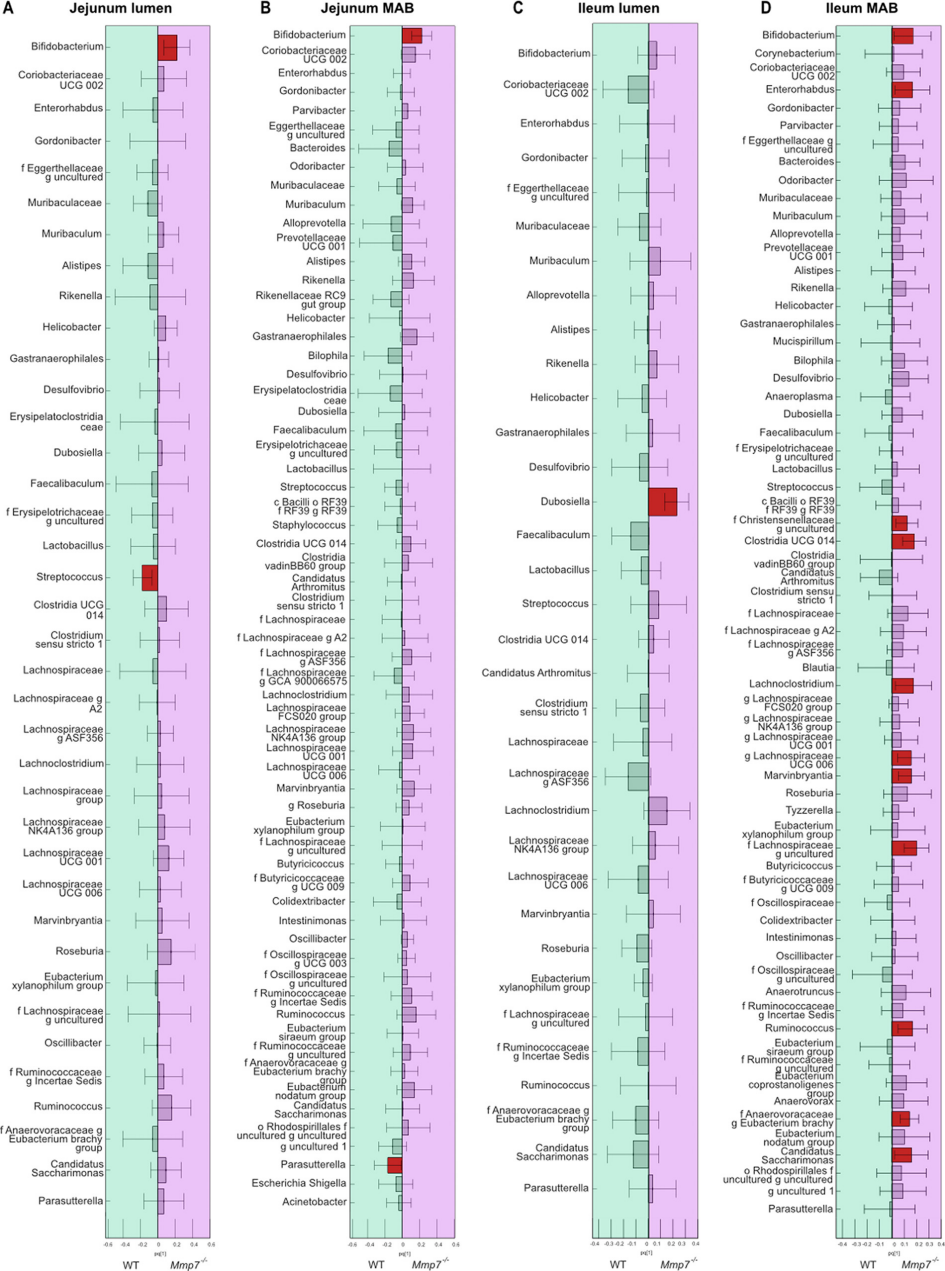
The abundance of distinct small-intestinal bacteria is modulated by defensins. (A) Orthogonal partial least squares discriminant analysis (OPLS-DA) of bacterial genera in the jejunal lumen, at the jejunal mucosa (B), in the ileal lumen (C), and at the ileal mucosa (D) of chow-fed WT and *Mmp7^−/−^* mice. A statistically significant difference in bacterial relative abundance shown by the red rectangles was defined when the confidence interval did not contain the null hypothesis value (0).

While diet caused a stronger shift in the overall bacterial composition than intestinal defensins, distinct changes in the abundance of luminal and mucosal taxa were also detected between genotypes. We thus performed a covariance analysis using an orthogonal partial least squares discriminant analysis (OPLS-DA) to disentangle in more detail which bacteria were affected by the presence of active defensins. Given the dominating impact of diet on microbiota composition, separate OPLS-DA were performed for chow-fed (Fig. 5, Fig. S3) and for WSD-fed mice (Fig. S4) for both jejunum and ileum.

Based on the OPLS-DA, *Bifidobacterium* was significantly enriched in the jejunal lumen and mucosa of chow-fed *Mmp7^−/−^* mice (Fig. 5A, B) compared to WT mice, which was supported by a direct comparison of *Bifidobacterium* abundance between the two genotypes (Fig. S3A, B). In addition, *Gastranaerophilales* was also enriched at the jejunal mucosa of the *Mmp7^−/−^* mice (Fig. S3B).

In the ileal lumen of chow-fed mice, only *Dubosiella* was enriched in the defensin-deficient mice compared to WT mice, based on both covariance (Fig. 5C) and relative abundance comparisons (Fig. S3C). At the ileal mucosa, the overall number of bacteria being enriched in the *Mmp7^−/−^* mice was higher than in the jejunum (Fig. 5D) and was significant for *Bifidobacterium*, *Dubosiella*, *Lachnoclostridium*, *Marvinbryantia*, [*Eubacterium*]*_brachy_group*, *Candidatus saccharimonas*, and *Lactobacillus* (Fig. 5D, Fig. S3D). Other taxa, including *Enterohabdus*, *Christensenellaceae*, *Clostridia_UCG_014*, *Ruminococcus*, and *Lachnospiraceae_UCG-006*, were significantly enriched in the *Mmp7^−/−^* mice in the OPLS-DA model (Fig. 5D), but this could not be confirmed when comparing relative abundances directly. In addition, and in contrast to the effect of diet observed at the ileal mucosa (Fig. 4H), *Candidatus arthromitus* was not affected by defensin deficiency under a chow diet (Fig. S3D).

When performing similar OPLS-DA of the bacterial composition with WSD-fed mice, the covariance analysis identified *Lactobacillus* as the single genus that was enriched at the jejunal mucosa as well as the jejunal and ileal lumen, but not at the ileal mucosa of WT mice (Fig. S4A to D). However, these findings were not confirmed when directly comparing the relative abundance of *Lactobacillus* between the two groups. Moreover, *Dubosiella* was enriched in the ileal lumen of WT mice fed the WSD (Fig. S4C). Accordingly, the WSD perturbation alone resulted in a shift of the microbial composition that was so robust that the lack of active defensins did not result in any major additional changes in the relative abundance of distinct taxa. These findings thus further confirm the dominating effect of the diet over defensins in shaping the small-intestinal bacterial community. Moreover, our results suggest that defensins might be required to maintain the abundance of specific genera of the ileal mucosa under regular dietary conditions.

### AMP expression is modulated by diet and defensin deficiency in the ileum.

**FIG 6:**
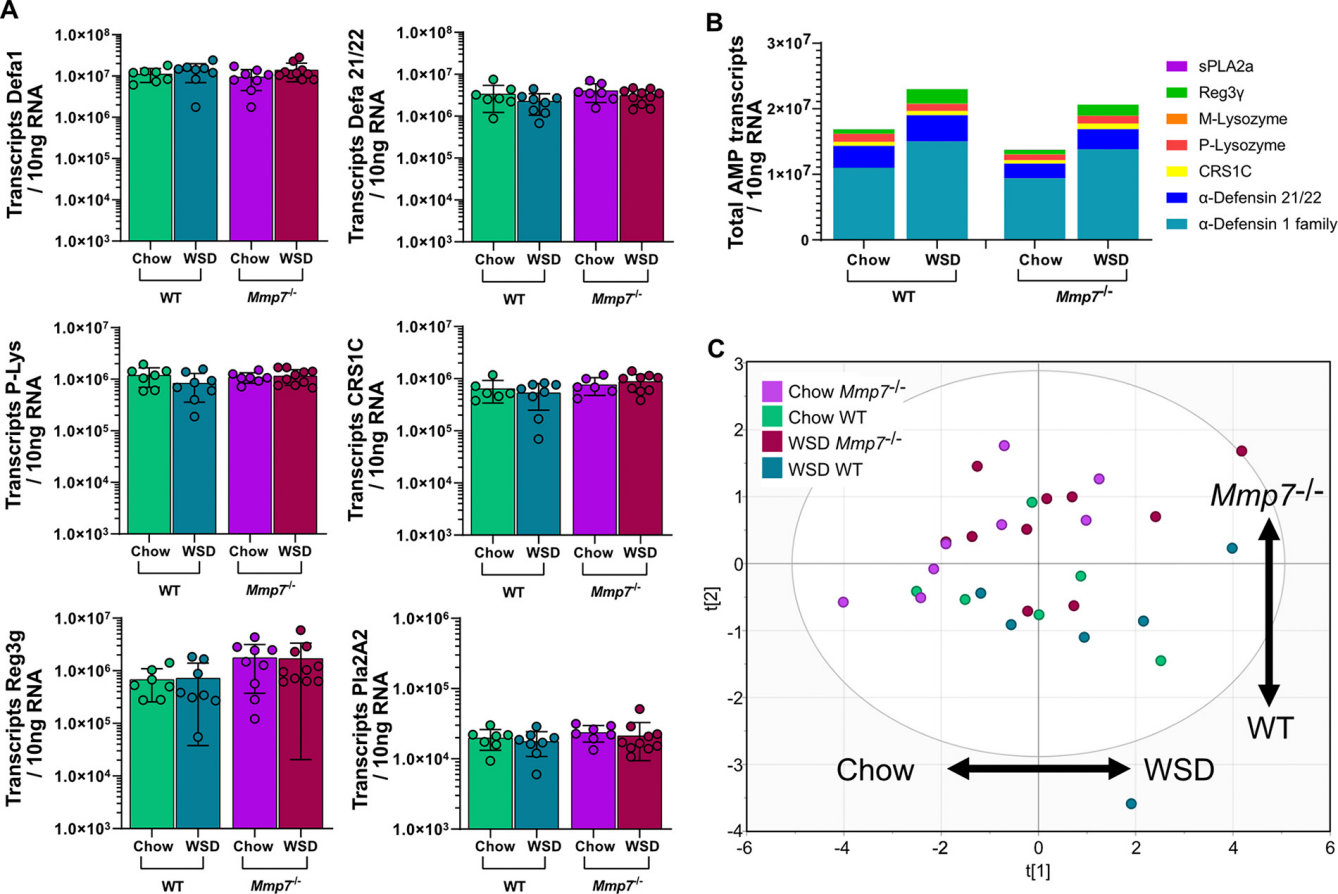
WSD feeding or defensin deficiency has a minor influence on AMP expression in the jejunum. (A) Expression of host-defense molecules Defa1, Defa21/22, P-Lysozyme (P-Lys), CRS1C, Reg3g, and Pla2a2 at the jejunum. (B) Sum of all measured AMP transcripts in the jejunum of the different mouse groups. (C) Principal-component analysis (PCA) of AMP expression at the jejunum of the different mouse groups. Statistical significance was determined by two-way ANOVA analysis with *, *P* ≤ 0.05; **, *P* ≤ 0.01; ***, *P* ≤ 0.001.

**FIG 7:**
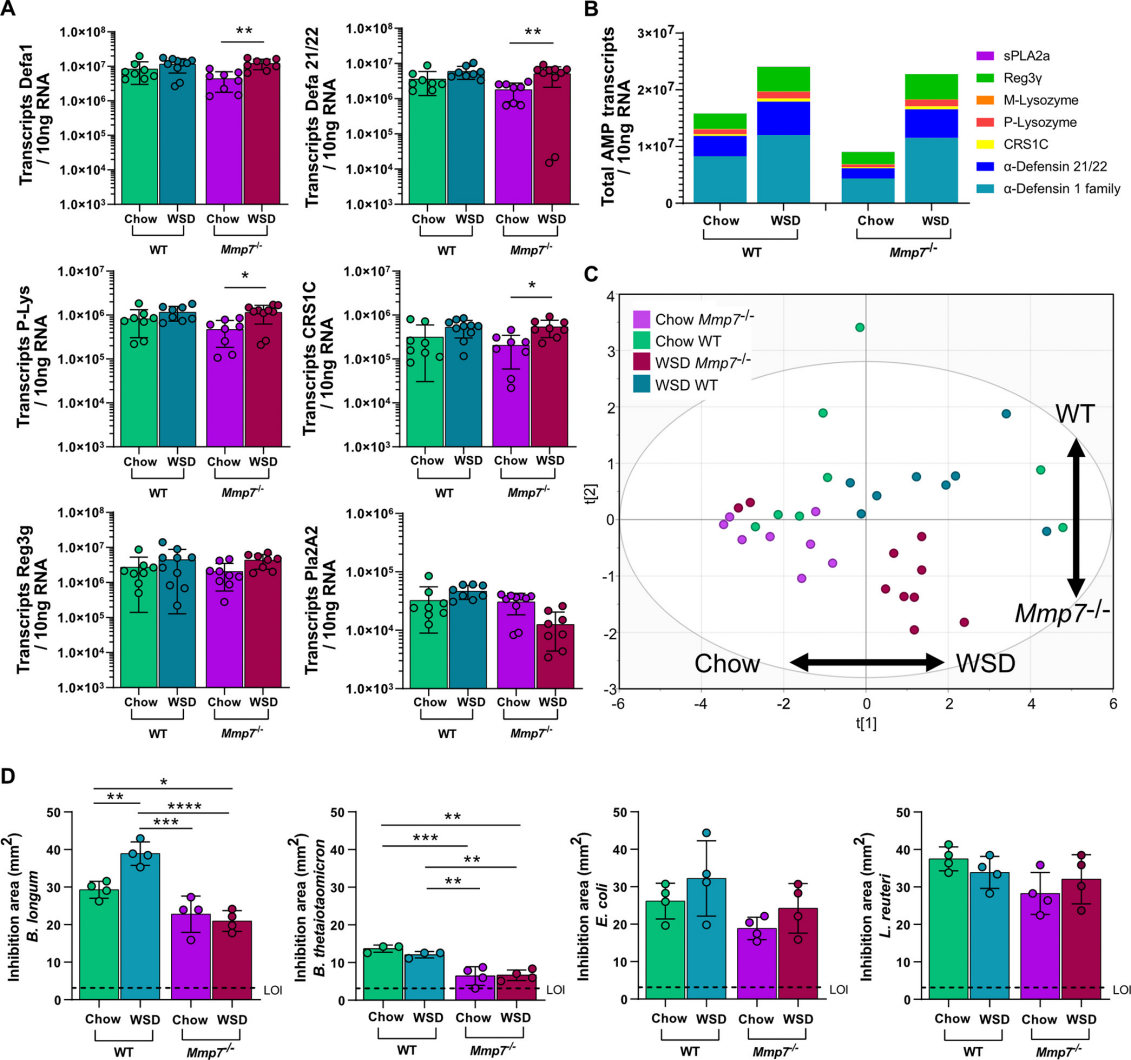
Diet is a substantial driver of AMP expression in the ileum. (A) Expression of host-defense molecules Defa1, Defa21/22, P-Lysozyme (P-Lys), CRS1C, Reg3g, and Pla2a2 at the ileum. (B) Sum of all measured AMP transcripts in the ileum of the different mouse groups. (C) Principal-component analysis (PCA) of AMP expression at the ileum of the different mouse groups. (D) Antimicrobial activity of ileal protein extract against Bifidobacterium longum, Bacteroides thetaiotaomicron, Escherichia coli, and Lactobacillus reuteri, by using a radial diffusion assay (RDA). Statistical significance was determined by two-way ANOVA analysis with *, *P* ≤ 0.05; **, *P* ≤ 0.01; ***, *P* ≤ 0.001. LOI, limit of inhibition.

Intrigued by the limited effect of defensin deficiency on small-intestinal bacterial composition, we reasoned that a compensatory upregulation of other AMPs could explain this effect. We thus investigated the specific impact of WSD and defensin deficiency on the expression of AMPs in the jejunum (Fig. 6A) and the ileum (Fig. 7A). By using absolute quantification of AMP transcripts and performing a multiple comparison analysis, we did not detect significant differences in the expression of the tested AMPs in the jejunum between any mouse groups (Fig. 6A). Since we quantified the absolute numbers of AMP transcripts, we were able to estimate the antimicrobial potential by summing the numbers of individual transcripts. Mice lacking functional defensins displayed a similar expression pattern as WT mice, and both groups had a tendency for increased AMP expression upon WSD feeding (Fig. 6B). However, when performing principal-component analysis (PCA) for AMP expression in the jejunum (Fig. 6C), no clear separation was detected based on diet (first component) or genotype (second component). Thus, jejunal AMP expression was not significantly affected by the absence of defensin function and was only modestly affected by the WSD. Moreover, this analysis revealed that the lack of defensins did not lead to an upregulation of other host-defense molecules such as Reg3g, Pla2A2, or P- or M-lysozyme at the jejunum.

Yet, in contrast to the jejunum, ileal expression of Defa1, Defa21/22, P-Lys, and CRS1C, but not Reg3g or Pla2A2, was significantly higher in the *Mmp7^−/−^* mice fed a WSD compared to chow-fed mice (Fig. 7A), while in WT mice only a tendency of increased expression for these Paneth cell products was observed. When summing the absolute transcript copy numbers in the ileum, *Mmp7^−/−^* mice had an overall decrease in AMP expression by ca. 43% in the chow-fed mice (Fig. 7B). Moreover, WSD treatment increased the overall AMP expression by ca. 52% in the WT mice and by ca. 152% in the defensin-deficient mice compared to the corresponding chow-fed groups. Accordingly, the PCA for AMP expression in the ileum showed a strong clustering based on diet (first component) and defensin deficiency (second component) (Fig. 7C), confirming the influence of both diet and genotype on AMP expression at the ileum.

Since higher AMP expression does not necessarily translate into higher antimicrobial activity, we next tested antibacterial activity of ileal protein extracts from the treated mice against selected commensal gut bacteria. In agreement with the increased expression upon WSD feeding, antimicrobial activity against B. longum increased in WSD-fed WT mice (Fig. 7D). Increased activity was however not observed in the *Mmp7^−/−^* extracts, confirming that, despite increased expression, the inactive defensins could not lead to increased killing of B. longum. These findings were further supported by a variable effect comparison test, in which the genotype, diet, and the interaction between diet and genotype all influenced the activity against B. longum (Table S3).

In agreement with previous studies (17), Bacteroides thetaiotaomicron (B. thetaiotaomicron) was rather resistant toward host-produced AMPs, indicated by low overall activity, which decreased even further in the absence of active defensins (Fig. 7D). Of note, and in contrast to the activity against B. longum, increased AMP expression upon WSD feeding did not result in higher antimicrobial activity in WT mice, suggesting that dietary modulation of antimicrobial activity is specific toward different microbial taxa. This was further confirmed by testing activity against E. coli and L. reuteri, for which no increased activity was observed upon WSD feeding. Similarly, a tendency for lower antimicrobial activity was observed in the absence of defensins, but these did not reach statistical significance (Fig. 7D). However, when performing a variable effect comparison test, only genotype significantly influenced the activity against B. thetaiotaomicron, E. coli, and L. reuteri (Table S3).

Altered antimicrobial activity against selected commensal bacteria after WSD feeding or in the absence of defensins could potentially affect the microbial load at the mucosa. Yet, the total bacterial 16S copy numbers at neither the mucosa nor lumen in the jejunum or ileum differed between WT and *Mmp7^−/−^* or between the two diets (Fig. S5). This is in agreement with a previous study (22) showing that defensin deficiency does not affect the overall ileal bacterial numbers. Taken together, both diet and defensin deficiency modulated AMP expression in the ileum, which resulted in distinct antimicrobial activity patterns that differed for individual bacterial strains.

### Combination of defensin deficiency and WSD aggravates glucose metabolism.

**FIG 8:**
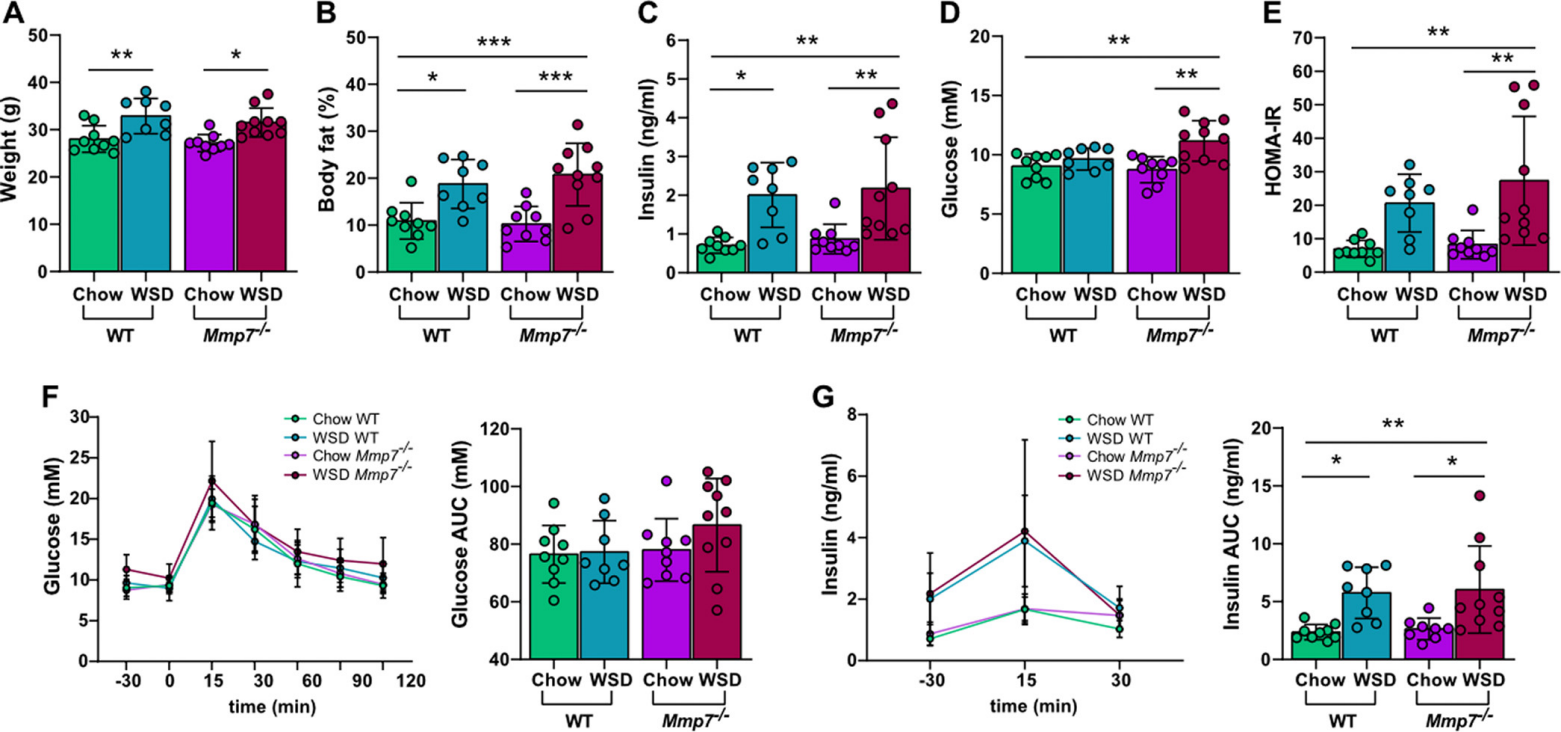
Combination of WSD and defensin deficiency exacerbates glucose metabolism. Defensin-deficient *Mmp7^−/−^* mice and WT mice were fed a chow or WSD for 8 weeks, after which (A) bodyweight, (B) body fat, (C) fasting blood insulin concentration, (D) fasting blood glucose concentration, and (E) homeostatic model assessment for insulin resistance (HOMA-IR) were determined. Two days prior to sacrifice, an oral glucose tolerance test (OGTT) was performed to determine (F) blood glucose and (G) insulin concentration and respective area under the curve (AUC). Statistical significance was determined by two-way ANOVA analysis with *, *P* ≤ 0.05; **, *P* ≤ 0.01; ***, *P* ≤ 0.001.

WSD consumption has been linked to microbiota-dependent development of obesity and metabolic diseases (36, 37), potentially due to the translocation of bacterial metabolites or intact bacteria across the intestinal mucosal barrier (38, 39). Since AMPs protect the host from close bacterial contact at the mucosa, we tested if defensin deficiency exacerbates metabolic impairments induced by WSD feeding. After 8 weeks of WSD feeding, mice displayed increased body weight (Fig. 8A), body fat (Fig. 8B), and insulin levels (Fig. 8C) compared to the chow-fed mice. These increases were independent of the genotype, but a stronger effect was observed in the defensin-deficient group. However, fasting blood glucose levels (Fig. 8D) and homeostatic model assessment for insulin resistance (HOMA-IR) values (Fig. 8E) were significantly higher only in the *Mmp7^−/−^* mice feeding on a WSD, indicating that the combination of defensin deficiency and WSD feeding worsens glucose metabolism (Table S4). These findings were further supported by an oral glucose tolerance test (OGTT), which displayed a tendency for impaired glucose tolerance (Fig. 8F) and higher insulin levels (Fig. 8G) in the WSD-fed *Mmp7^−/−^* mice. Thus, defensin deficiency is not sufficient to cause metabolic impairments upon feeding a control diet, but in combination with a high-caloric WSD, metabolic parameters are aggravated, particularly glucose metabolism.

## DISCUSSION

The microbiota composition in a defined host-associated environment is a product shaped by different intrinsic and external parameters. In the gastrointestinal tract, several niches exist that differ in structural architecture and physiological function, thereby varying in pH, nutrient availability, and presence of antimicrobials, as well as oxygen concentration (40). We here disentangled the factors contributing to the jejunal and ileal bacterial composition in the lumen and at the mucosa. We observed that the most substantial effect on bacterial composition was exerted by the intake of a WSD, which is in agreement with a myriad of studies implicating diet as the most potent modulator also for the colonic gut microbiota composition (25, 41, 42).

Already in the 1980s, Rolf Freter formulated in his nutrient-niche hypothesis that the ability of a microbe to establish itself in the gut environment will largely depend on its ability to most efficiently use a limited nutrient available at a particular niche (43, 44). Dietary fibers are an important nutrient source for many commensal gut bacteria, and consequently their absence in the lumen through WSD consumption forces generalist commensal bacteria to relocate to the carbohydrate-rich mucus layer instead. Moreover, the abundance of fiber specialist commensals will be reduced, and accordingly the WSD will cause community changes in both the luminal and mucosal environments (45). However, and extending Freter’s niche hypothesis, diet can modulate the microbial community not only by providing substrates, but also through directly affecting the ecological niche. As such, we recently showed that the jejunal small intestinal mucus provides a niche to the mucosal microbiota, which mediated colonization resistance against the intestinal pathogen Citrobacter rodentium (46). Importantly, WSD feeding changed the structure and integrity of the jejunal mucus layer, which thus lost its ability to provide a niche to the commensal microbiota, consequently leading to atypical colonization of this niche by C. rodentium. Therefore, diet composition and nutrient availability are key factors determining which microbes can colonize the different intestinal niches and thus become indigenous residents of the gut microbiota.

Besides diet, defensins have been reported to modulate the small intestinal microbiota composition in two complementary mouse models (47). In that landmark study, 5-week-old transgenic mice expressing the human alpha-defensin HD5 in addition to their intrinsic defensin repertoire showed increased relative abundance of Bacteroidetes and a decrease in Firmicutes abundance in the ileum. Conversely, young defensin-deficient *Mmp7^−/−^* mice exhibited an increase in Firmicutes and a decrease in Bacteroidetes (22). The intestinal bacterial composition in mice has been described as stabilizing after the age of 6 weeks (48), and it is thus possible that defensins are essential in shaping the small intestinal bacterial composition in these first weeks of life, as described by Salzman et al. (22). However, in our study using 16-week-old mice, we did not observe major changes in phyla or genus composition in the small intestine of *Mmp7^−/−^* mice (22), which is in agreement with previous studies using the same defensin-deficiency mouse model (23, 24).

In addition to the age difference, the inherent intestinal microbiota composition may determine the susceptibility or resilience toward community disturbances. As such, Salzman et al. (22) obtained their mice from a different animal provider than us and the other two mentioned studies (23, 49). Since mice from different vendor laboratories exhibit a different microbial community (35, 50), it is possible that a distinct baseline microbiota responds differently to disturbances in defensin function, especially once the community has matured. Lastly, while Salzman et al. used 16S subclone sequencing on an Applied Biosystems capillary sequencer, we performed total 16S sequencing of the V4 region on an Illumina MiSeq machine and analyzed the amplicons by using the latest reference databases. As microbial databases have been continuously refined since 2010 and several taxa have been regrouped, it is possible that changes in the reference databases may also contribute to the opposing outcomes. Unfortunately, the absence of original samples from previous studies makes it impossible to directly compare our results with those of the previous literature. Further studies at different geographical locations with identical methodological setup would thus be required to conclusively identify whether the different results based on the presence or absence of defensins are rather of technical or of biological reason. It should be noted, however, that Mastroianni et al. identified an additional mechanism of defensin activation in the colon by human and bacterial proteases in the *Mmp7^−/−^* mice (49). Nevertheless, this alternative activation did not influence defensin maturation in the small intestine and was restricted to the colon, thus explaining the similar bacterial composition in the colon, but not in the small intestine, of *Mmp7^−/−^* mice (49).

Despite the lack of major changes on the phylum level, we found that chow-fed WT and *Mmp7^−/−^* mice clustered separately in the Bray-Curtis distance analysis in the jejunal and ileal lumen and that several genera were enriched at the ileal mucosa of the chow-fed *Mmp7^−/−^* mice. Increased abundance of these genera at the mucosa suggests that defensins would kill these microbes under normal conditions. This interpretation would be in agreement with previous analysis on the host-defense molecule Reg3g (51), in which SFB were enriched at the ileal mucosa of *Reg3g^−/−^* mice. Furthermore, a recent study that evaluated the impact of a high-fat diet and Reg3g on microbiota composition identified diet as the stronger microbiota-modulating factor (52), while in this mouse model even the absence of Reg3g led to differential bacterial clustering upon chow diet feeding, based on PCA analysis.

In our analysis of defensin-deficient mice, we found increased abundance of *Dubosiella* in the ileum lumen and mucosa. *Dubosiella* is a Gram-positive rod belonging to the family Erysipelotrichaceae that was first isolated from mouse intestinal lumen (53). Recently, mice deficient in the AMP lipocalin-2 and fed a high-fat diet for 12 weeks were shown to have enriched fecal levels of *Dubosiella* (54). While the authors suggested that *Dubosiella* might play a detrimental role in obesity-related microbial dysbiosis (54), other studies suggested that it is a potential probiotic candidate for beneficial modulation of total fat mass and intestinal immunity (55, 56). However, in our study we did not observe any enrichment of *Dubosiella* in the small intestine of the *Mmp7^−/−^* mice during WSD feeding (Fig. S4), which could be explained by different dietary sources (high-fat versus WSD), differences in the length of the dietary intervention, or different antimicrobial activity between lipocalin-2 and the large group of defensins. Yet, the links between *Dubosiella*, antimicrobial peptides, and metabolic phenotypes warrant further investigation.

When measuring AMP transcripts, we observed that WSD feeding led to higher expression of total AMPs in the jejunum and ileum compared to chow-fed mice. While these findings are supported by some previous studies (57, 58), they are also contradicted by others (31, 59), and there are several aspects that might explain the discrepancy. First, the regulation of AMP expression is not yet entirely understood and seems to be mediated by the development process (60), the mTOR nutrient-sensing pathway (61), lipopolysaccharide (LPS) (24), and short chain fatty acids (SCFAs) (31, 62), and influenced by the circadian cycle (52). Second, the description of age, sex, and littermate matching of the mouse models is often not reported in the literature, making it difficult to directly compare studies. Third, the inherent microbiota of different animal facilities can additionally influence which microbes respond to the diet intervention and consequently influence AMP expression. Indeed, a recent study found that out of three different taxa that positively correlated with small intestinal *Reg3g* expression in mice, only the metabolites produced by Lactobacillus rhamnosus LGG were capable of inducing *Reg3g* expression in small intestinal enteroids (52). Consequently, regulation of AMP expression is complex, and different microbial factors may have contributed to the observed increase in AMP expression after the WSD intake in this and other studies.

Mice deficient in active defensins and fed a WSD had impaired metabolic parameters compared to WT mice. High-fat diets have been shown to increase bacterial translocation across the mucosal barrier, leading to obesity-associated inflammation caused by endotoxemia in mice (38, 63–66) and potentially even in humans (67, 68). Furthermore, in accordance with our findings on increased AMP expression upon WSD feeding, Guo et al. observed that feeding mice with a high-fat diet resulted in an overall increased expression of several AMPs and led to increased inflammation and endoplasmic reticulum stress (57). Increased AMP expression correlated positively with plasma LPS and inflammatory mediators (57), thus suggesting that this upregulation could arise as a compensatory mechanism to protect against bacterial translocation. This is further supported by a study in *Myd88^−/−^* mice, which display a defective mucosal antimicrobial peptide response and had increased intestinal translocation of both commensal and pathogenic bacteria (15). While we did not measure penetrability of intestinal bacteria in our study, it is possible that the WSD-fed *Mmp7^−/−^* mice had increased bacterial translocation compared to the WT and chow-fed mice. Consequently, the absence of active defensins could have exacerbated the WSD-induced dysbiosis and bacterial translocation in the small intestine and thereby contributed to the metabolic impairments.

Of note, Mmp7 has recently been shown to degrade the tight junction protein claudin-7 in the colon of a mouse model of colitis, leading to increased bacterial translocation (69). As a lack of Mmp7 would in such a case rather be protective, it is unlikely that removal of Mmp7 in the colon is involved in the decreased barrier function and increased bacterial translocation. In addition, as a member of the matrix metalloproteinase family that is typically involved in extracellular matrix degradation, Mmp7 function has been linked to tissue repair (70, 71), transepithelial influx of neutrophils (72), and platelet activation during uremia (73). Thus, we cannot fully rule out any potential intestinal effects that the absence of Mmp7 may have in the observed metabolic defects. Yet, no metabolic defects were observed in the *Mmp7^−/−^* mice fed the control diet, highlighting that the metabolic impairments only occurred in combination of defensin deficiency with the intake of a WSD.

In conclusion, our results consistently show that diet is the major modulator of the small intestinal bacterial composition and that intestinal defensins rather play a minor role. However, the protective function of AMPs becomes more relevant in the face of dysbiosis-inducing challenges, such as WSD feeding, where they contribute to an active mucosal defense that limits the development of metabolic impairments, or antibiotic intervention, where α-defensins were shown to support the recovery and mucosal colonization of the genus *Bacteroides* (23). Yet, although the effect of defensins on the bacterial composition was not comparable to the WSD, we demonstrate that defensins fine-tune the composition in the small intestinal mucosa, highlighting their intricate spatial regulation of the microbial community in the intestine.

## MATERIALS AND METHODS

### Mice.

Mice were cohoused with up to 5 mice/cage under specific pathogen-free (SPF) conditions with a 12-h light/dark cycle and had unlimited access to water and food. All mouse experiments were approved by the University of Gothenburg, Sweden. Male Mmp7-deficent mice (B6.129-Mmp7tm1Lmm/J; *MMP7^−/−^*) were originally purchased from Jackson Laboratory, USA, and crossed with in-house-bred wild-type (WT) female C57BL/6 mice. The generated heterozygous litters were used to produce *Mmp7^−/−^* animals as well as corresponding wild-type littermates. Mmp7 status was verified via genotyping, and mice were separated according to genotype after weaning to prevent microbiota exchange via coprophagy. At the age of 8 weeks, WT and *Mmp7^−/−^* mice either were switched to a Western-style diet (TD.96132 Envigo) or remained on a control chow diet (5021 LabDiet) for another 8 weeks. The mice were sacrificed by cervical dislocation after anesthesia with isofluorane.

### DNA extraction and 16S rRNA sequencing library generation.

The small intestine (SI) was dissected, divided into 8 equal parts, and numbered from proximal to distal segments. The fifth segment is considered to represent the jejunum, and the eighth represents the ileum. Following dissection, tissue pieces were immediately snap-frozen in liquid nitrogen. Genomic DNA from mucosal tissue and intestinal lumen contents was extracted by repeated bead-beating using lysis buffer (4% [wt/vol] SDS, 50 mM Tris HCl pH 8, 500 mM NaCl, 50 mM EDTA) and a Fast-Prep System with Lysing Matrix E (MPBio) as described previously (74).

The extracted and purified DNA was used for library preparation for 16S rRNA sequencing on an Illumina MiSeq machine. Briefly, the V4 region of the 16S rRNA gene was amplified using 515F and 806R primers designed for dual indexing (75), and the amplicons were sequenced with a V2 kit (2 × 250 bp paired-end reads) on the MiSeq machine. The amplification was controlled for purity with a nontemplate control for each amplicon corresponding to each sample. Lumen content samples were amplified in duplicate, while mucosa-associated bacteria (MAB) tissue samples were amplified in triplicate in a reaction volume of 25 μL containing 100 ng of genomic DNA, 1× Five Prime Hot Master Mix (Quantabio), 0.2 μM final of each primer, 0.4 mg/mL bovine serum albumin (BSA) and 5% DMSO (lumen), or 0.8 mg/mL BSA and 10% DMSO (MAB). PCR amplification was performed as follows: initial denaturation at 94°C for 3 min, 25 cycles (lumen samples) or 26 cycles (MAB samples) of denaturation at 94°C for 45 s, annealing at 52°C for 60 s and elongation at 72°C for 90 s, and a final elongation step at 72°C for 10 min. The PCR products were purified using the NucleoSpin Gel and PCR Clean-Up kit (Macherey-Nagel), quantified (Quant-iT PicoGreen dsDNA kit; Thermo Fisher Scientific), and pooled to equimolar amounts. The pooled 16S amplicons were further purified using Mag-Bind magnetic purification beads (Omega Biotek) before denaturation of the libraries for loading into the Illumina V2 cartridge following sequencing on an Illumina MiSeq system.

### 16S rRNA sequencing analysis.

The generated fastq files were processed using Qiime2 (v.2019.10) (76). Adapter sequences were trimmed, demultiplexed, and quality filtered followed by denoising with DADA2 (77). Taxonomy was assigned using the SILVA v138-515f-806r classifier (78) to generate amplicon sequencing variants (ASVs). The data were further filtered to remove mitochondria and chloroplasts, and specific Lactococcus lactis ASVs were removed since it is a known contaminant in the WSD pellets (79). A phylogenetic tree of the sequences was created with the MAFFT software v.7.407 (80), and low abundant sequences (relative abundance <0.002%) were removed. Αlpha diversity (observed ASVs and Shannon index) and beta diversity (Bray-Curtis distance, unweighted UniFrac, and weighted UniFrac) were performed after rarefaction (2,390 reads for jejunum lumen, 5 samples excluded; 5,600 reads for ileum lumen, 2 samples excluded; 1,900 for jejunum MAB, 6 samples excluded; and 2,000 for ileum MAB, 4 samples excluded). The relative abundance at the phylum and genus level and alpha and beta diversity analysis were plotted in Prism 9.3.1 (GraphPad Software, Inc.).

### RNA extraction and cDNA generation.

Directly after sacrifice, small biopsy specimens from the mouse jejunum and ileum were collected, snap-frozen in liquid nitrogen, and stored at –80^0^C. For RNA extraction, the tissue was homogenized with stainless steel beads (5 mm) (Qiagen) in a TissueLyser II (Qiagen), and RNA was extracted using a RNeasy minikit (Qiagen). The RNA quantity and quality were assessed by NanoDrop (Thermo Fisher Scientific). A total of 500 ng of RNA was reverse transcribed into cDNA with a High-Capacity cDNA Reverse Transcription kit (Thermo Fisher Scientific) and diluted 1:7 in nuclease-free water.

### Transcript quantification by RT-qPCR.

Mouse cDNA was amplified with gene-specific primers (Table 1) (60) and a HotStarTaq Master Mix kit (Qiagen). Amplicons were cloned into pGEM-T vector (Promega) and transformed into One Shot MAX Efficiency DH10B T1 cells (Invitrogen). Plasmids were isolated (Qiagen Plasmid minikit), sequenced (Eurofins Genomics, Ebersberg, Germany), quantified, and diluted in series of 10. Jejunal and ileal samples were analyzed in a 10-μL reaction mix consisting of 1× iQ SYBR Green Supermix (Bio-Rad), 0.2 μM each primer, and 2 μL of template cDNA on a CFX Connect Real-Time System (Bio-Rad). Amplification of samples and plasmid standards was performed as follows: denaturation at 95°C for 3 min, followed by 35 cycles of denaturation at 95°C for 20 s, gene-specific annealing temperature (Table 1) for 40 s, and extension at 72°C for 60 s. A standard curve was generated, and the transcript copy number in each sample was calculated using the Bio-Rad CFX maestro software and reported as copy number/10 ng RNA.

**Table 1:** Primer sequences used in this study and annealing temperature used for qPCR

Name	Product	Primer sequence		Annealing temperature	Reference
Alpha defensin 1 family	Defa1	Fwd (5′-3′)	TCAAGAGGCTGCAAAGGAAGAGAAC	63°C	60
		Rvs (5′-3′)	TGGTCTCCATGTTCAGCGACAGC		
Alpha defensin 21/22	Defa21/22	Fwd (5′-3′)	CCAGGGGAAGATGACCAGGCTG	63°C	60
		Rvs (5′-3′)	TGCAGCGACGATTTCTACAAAGGC		
Cryptdin related sequence (CRS) peptides Group 1C	CRS1C	Fwd (5′-3′)	CACCACCCAAGCTCCAAATACACAG	68°C	60
		Rvs (5′-3′)	ATCGTGAGGACCAAAAGCAAATGG		
Reg III gamma	Reg3g	Fwd (5′-3′)	CCTCAGGACATCTTGTGTCTGTGCTC	68°C	60
		Rvs (5′-3′)	TCCACCTCTGTTGGGTTCATAGCC		
Paneth cell specific lysozyme	P-Lysozyme	Fwd (5′-3′)	GCCAAGGTCTACAATCGTTGTGAGTTG	66°C	60
		Rvs (5′-3′)	CAGTCAGCCAGCTTGACACCACG		
Myeloid-specific lysozyme	M-Lysozyme	Fwd (5′-3′)	GGCTGGCTACTATGGAGTCAGCCTG	65°C	60
		Rvs (5′-3′)	GCATTCACAGCTCTTGGGGTTTTG		
Secretory phospholipase A2	sPLA2a	Fwd (5′-3′)	AGGATTCCCCCAAGGATGCCAC	68°C	60
		Rvs (5′-3′)	CAGCCGTTTCTGACAGGAGTTCTGG		
Matrix metalloproteinase 7	Mmp7	Fwd (5′-3′)	TTCAAGAGGGTTAGTTGGGGGACTG	65°C	60
		Rvs (5′-3′)	TTGTCAAAGTGAGCATCTCCGCC		
16S rDNA	Total bacteria	Eub338F (5′-3′)	ACTCCTACGGGAGGCAGCAG	60°C	81
		Eub518R (5′-3′)	ATTACCGCGGCTGCTGG		

### Orthogonal partial least-squares discriminant analysis.

Orthogonal partial least-squares discriminant analysis (OPLS-DA, performed in Simca 16.0 [Sartorius Stedim Data Analytics, Sweden]) was applied to identify differences in the abundance of individual bacterial taxa between the two diets or two genotypes.

Prior to modeling, a filtering step was performed to effectively remove orthogonal variation and to only keep predictive variation in the model. The loading vector was equal to the direction in the multivariate space that discriminates between WT and *Mmp*7^−/−^ groups to the greatest extent, and the loading vectors were suitable for interpretation of the difference between the WT and *Mmp*7^−/−^ samples. Positive loadings correlated positively to the *Mmp*7^−/−^ samples, while the corresponding taxa were of higher abundance in this group. Larger loading values indicated a higher influence on the model and as such, a more pronounced difference between WT and *Mmp*7^−/−^ for the different taxa. Significantly regulated bacteria in the OPLS-DA were detected when the confidence interval did not contain the null hypothesis value (0).

### Intestinal protein extraction.

Ileal biopsy specimens were mixed with extraction buffer (60% acetonitrile, 1% trifluoroacetic acid) and disrupted in a Tissue Lyser II (Qiagen) at 25 Hz for 30 s using a stainless-steel bead (5 mm, Qiagen). The same program was repeated every 20 min for 2 h, keeping the tissue at 4°C between disruption cycles. The samples were centrifuged for 20 min at 4°C at 18,000 rcf (relative centrifugal force), and dried in a SpeedVac at low temperature. The obtained intestinal protein powder was resuspended in 0.01% acetic acid, and the protein concentration was determined with a Pierce BCA protein assay kit (ThermoFisher), following the manufacturer’s protocol.

### Radial diffusion assays.

Escherichia coli DSM 301, Lactobacillus reuteri DSM 20016, Bifidobacterium longum NCC 2705 (Nestec), and Bacteroides thetaiotaomicron DSM 2079 were grown in Trypticase soy broth (TSB) 30 g/L (BD); De Man, Rogosa, and Sharpe (MRS) 51 g/L (Sigma-Aldrich); reinforced clostridium medium (RCM) 37.5 g/L (VWR); and brain heart infusion supplemented with 5g/L yeast extract, 0.5 mg/L hemin, 0.2g/L NaHCO_3_, and 1g/L cysteine (BHI-S), respectively, at 37°C. E. coli and L. reuteri were incubated aerobically, and B. longum and B. thetaiotaomicron were incubated under anaerobic conditions (0% O_2_, 5% CO_2_, 10% H_2_, and 85% N_2_) in an anaerobic chamber (Don Whitley Scientific, UK), until reaching exponential phase. Subsequently, bacteria were washed and resuspended in sterile ice-cold 10 mM sodium phosphate buffer (pH 7.4), and approximately 1 × 10^7^ CFU were added to 10 mL of a lukewarm underlay gel consisting of 0.1% EEO-Agarose (Sigma), 0.1% TSB, MRS, RCM, or BHI-S broth, and 10 mM sodium phosphate buffer adjusted to pH 7.4, and poured into an empty petri dish. After the gel solidified, 2-mm-diameter holes were punched into the agar, and 4 μL of a 1-μg/μL intestinal peptide extract was added into the holes. The underlay gel was incubated for 3 h to allow the diffusion of the intestinal extract into the gel. After the 3-h incubation, 10 mL of overlay gel, consisting of 3% TSB, MRS, RCM, or BHI-S broth powder, 0.1% EEO-Agarose (Sigma), and 10 mM sodium phosphate buffer adjusted to pH 7.4, were added on top of the underlay gel. The gel was incubated at 37°C under aerobic or anaerobic conditions for 24 h, after which the diameter of inhibition zone of each extract was measured. Antimicrobial activity was expressed as inhibition area and calculated by multiplying the square radius of the inhibition with π. The experiments were performed in duplicate for each bacterium.

### Total bacteria quantification by RT-qPCR.

Absolute bacterial 16S gene copy numbers were quantified using a custom-made plasmid containing a 16S gene amplicon from *E. coli* DSM 301. The 16S gene was amplified from a colony of E. coli grown in LB agar, in a reaction mixture containing 10 μL HotStartTaq master mix (Qiagen), 0.2 μM universal primers (Eub338F and Eub518R, Table 1), and 100 ng of template DNA in a final 20 μL reaction volume with the following PCR program: denaturation at 95°C for 5 min, followed by 35 cycles of denaturation at 94°C for 30 s, annealing at 55°C for 30 s, and extension at 72°C for 60 s and extension at 72°C for 10 min. The amplicon was purified with the E.Z.N.A Cycle Pure kit (Omega), cloned into a pGEM-T vector (Promega), and transformed into E. coli DHα electrocompetent cells. The plasmid was isolated with the Plasmid minikit (Qiagen) and sequenced (Eurofins Genomics, Ebersberg, Germany), and a standard curve based on 16S gene copies was generated.

16S gene copy number in the mouse samples was determined by amplification of the 16S rRNA gene on a CFX Connect Real-Time System (Bio-Rad). The reaction mix consisted of 1 × SYBR Green Master Mix (Bio-Rad), 0.2 μM universal primers Eub338F and Eub518R, and 2 ng of DNA template with the following RT-qPCR program: denaturation at 95°C for 3 min, followed by 35 cycles of denaturation at 95°C for 15 s, and annealing at 60°C for 30 s and extension at 72°C for 30 s. The absolute bacterial 16S copy number in each sample was quantified using 10-fold serially diluted standard curves generated with the custom-made plasmid.

### Metabolic measurements.

Mice were fasted for 4 h, and glucose concentration was measured in tail vein whole blood with commercial blood glucose strips (Contour Next, Bayer). Insulin concentration was determined in serum by Ultra Sensitive Mouse Insulin ELISA kit (Crystal Chem). Body fat was determined by whole-body magnetic resonance imaging (EchoMRI, Echo Medical Systems). The homeostatic model assessment insulin resistance index (HOMA-IR) was determined with the formula HOMA-IR = (insulin (mU/L) * glucose (mM))/22.5. Oral glucose tolerance test (OGTT) was performed by gavaging fasted mice with a glucose solution (2 mg/g body weight). Blood samples were collected from tail vein at 0, 15, 30, 60, 90, and 120 min, and blood glucose and insulin levels were determined as described above.

### Statistical analysis.

For comparisons of nonparametric data between more than two groups, a two-way ANOVA test was done in Prism 9.3.1 (GraphPad Software, Inc.). Error bars in the graphs represent standard deviation. In Fig. 1G, I; 2F, H; 3F, H; 4F, H; 7D; and 8A to G, a two-way ANOVA test was performed to determine the % of variance explained by the independent variables diet, genotype, or their interaction on the corresponding dependent variables, and is summarized in Tables S1 to S4, followed by a Tukey’s multiple-comparison test. Statistical group differences in the microbial diversity analyses were tested with a pairwise Kruskal-Wallis test for alpha diversity analysis and with a permutational analysis of variance (PERMANOVA) test with 999 permutations for the beta diversity analysis.

### Data availability.

Amplicon sequences have been deposited in the European Nucleotide Archive (https://www.ebi.ac.uk/ena/) with accession number PRJEB59609.
