# Supplementary material for: Intestinal α-Defensins Play a Minor Role in Modulating the Small Intestinal Microbiota Composition as Compared to Diet
Source: Microbiol Spectr. 2023 Apr 11;11(3):e00567-23. doi: 10.1128/spectrum.00567-23 (PMC10269482; doi:10.1128/spectrum.00567-23)
Supplement: Supplemental file 1 — Supplemental material. Download spectrum.00567-23-s0001.pdf, PDF file, 1.3 MB [file spectrum.00567-23-s0001.pdf]

Supplementary Table 1. Effect of diet or genotype on bacteria relative abundance by 2-way ANOVA analysis.

Jejunum content

	Diet		Genotype		Interaction	
Phylum or genera relative abundance	% variation	p-value	% variation	p-value	% variation	p-value
Firmicutes	60.13	**<0.0001**	1.44	0.2714	2.53	0.1491
Bacteroidetes	65.66	**<0.0001**	0.60	0.4832	0.05	0.8285
Actinobacteria	7e-3	0.9580	0.69	0.6219	20.25	**0.0118**
Bifidobacterium	0.50	0.6680	3.24	0.2832	17.32	**0.0173**
Coriobacteriaceae_UCG-002	4.84	0.2338	0.24	0.7859	0.09	0.8628
Streptococcus	0.74	0.6293	2.82	0.3492	5.91	0.1790
Lactobacillus	0.93	0.5748	11.53	0.0557	3.15	0.3060
Faecalibaculum	60.88	**<0.0001**	7e-3	0.9368	2.88	0.1186
Muribaculaceae	65.03	**<0.0001**	0.58	0.4943	0.06	0.8129

Ileum content

	Diet		Genotype		Interaction	
Phylum or genera relative abundance	% variation	p-value	% variation	p-value	% variation	p-value
Firmicutes	73.57	**<0.0001**	7.1e-5	0.9931	0.02	0.8826
Bacteroidetes	79.91	**<0.0001**	0.01	0.8644	0.05	0.7759
Actinobacteria	2.37	0.4087	0.63	0.6683	2.30	0.4153
Bifidobacterium	0.03	0.9245	0.44	0.7187	5.43	0.2133
Coriobacteriaceae_UCG-002	34.06	**0.0005**	0.45	0.6536	2.50	0.2982
Streptococcus	5.59	0.1962	2.06	0.4285	3.66	0.2930
Lactobacillus	0.13	0.8408	4.44	0.2600	1.75	0.4762
Faecalibaculum	57.77	**<0.0001**	1.65	0.2131	10.81	**0.0030**
Muribaculaceae	77.50	**<0.0001**	6e-3	0.9200	0.04	0.8067

Supplementary Table 2. Effect of diet or genotype on bacteria relative abundance by 2-way ANOVA analysis.

Jejunum MAB

Phylum or genera relative abundance	Diet		Genotype		Interaction	
	% variation	p-value	% variation	p-value	% variation	p-value
Firmicutes	64.11	**<0.0001**	3.68	0.1010	6.68	**0.0306**
Bacteroidetes	84.20	**<0.0001**	5e-3	0.9244	2.07	0.0822
Actinobacteria	11.53	**0.0413**	27.55	**0.0028**	1.66	0.4211
Bifidobacterium	2.87	0.3552	18.73	**0.0241**	0.04	0.9089
Gastranaerophilales	15.13	**0.0079**	15.13	**0.0079**	15.13	**0.0079**
Parasutterella	74.08	**<0.0001**	1.65	0.1796	1.62	0.1834
Lactobacillus	2.96	0.3364	7.23	0.1380	7.47	0.1318
Faecalibaculum	42.82	**0.0002**	0.07	0.8585	0.08	0.8481
Muribaculaceae	53.09	**<0.0001**	2.85	0.2167	0.25	0.7077

Ileum MAB

Phylum or genera relative abundance	Diet		Genotype		Interaction	
	% variation	p-value	% variation	p-value	% variation	p-value
Firmicutes	27.19	**0.0016**	2.13	0.3398	0.44	0.6605
Bacteroidetes	55.05	**<0.0001**	1.32	0.3272	1.79	0.2560
Actinobacteria	11.59	**0.0425**	6.28	0.1295	1.36	0.4728
Bifidobacterium	3.35	0.2854	4.39	0.2225	1.11	0.5363
Coriobacteriaceae_UCG-002	34.60	**0.0002**	1.37	0.4088	0.02	0.9080
Dubosiella	0.03	0.9124	2.24	0.3935	1.87	0.4349
Lachnoclostridium	1.80	0.3983	2.78	0.2949	16.30	**0.0148**
Marvinbryantia	24.60	**0.0006**	10.93	**0.0168**	9.37	**0.0259**
[Eubacterium]_brachy_group	16.86	**0.0067**	9.30	**0.0391**	9.30	**0.0391**
Lactobacillus	8.73	0.0619	6.48	0.1052	12.07	**0.0298**
Faecalibaculum	58.08	**<0.0001**	0.92	0.3722	2.28	0.1650
Muribaculaceae	54.18	**<0.0001**	1.26	0.3337	1.87	0.2412
Candidatus_Arthromitus	23.47	**0.0028**	2.30	0.3181	1.11	0.3243

Supplementary Table 3. Effect of diet or genotype on the activity of the protein extract 2-way ANOVA analysis.

Transcript	Diet		Genotype		Interaction	
	% variation	p-value	% variation	p-value	% variation	p-value
Escherichia coli	14.61	0.1111	26.09	**0.0403**	0.04	0.9238
Bifidobacterium longum	6.58	**0.0399**	64.39	**<0.0001**	14.15	**0.0055**
Lactobacillus reuteri	7e-3	0.9713	25.14	**0.0499**	11.38	0.1681
Bacteroides thetaiotaomicron	1.00	0.4506	81.45	**<0.0001**	1.63	0.3389

Supplementary Table 4. Effect of diet or genotype on metabolic parameters by 2-way ANOVA analysis.

Transcript	Diet		Genotype		Interaction	
	% variation	p-value	% variation	p-value	% variation	p-value
Weight (g)	40.75	**<0.0001**	2.39	0.2578	0.12	0.7978
Glucose (nM)	24.25	**0.0011**	4.04	0.1516	8.83	**0.0375**
Insulin (ng/ml)	40.07	**<0.0001**	0.64	0.5554	3e-4	0.9891
HOMA-IR	35.47	**0.0001**	2.11	0.2949	0.96	0.4778
Body fat (%)	46.68	**<0.0001**	0.24	0.6944	0.97	0.4389
Glucose AUC (nM)	3.53	0.2665	4.67	0.2027	2.44	0.3539
Insulin (AUC)	36.95	**0.0001**	0.27	0.7096	2e-4	0.9911

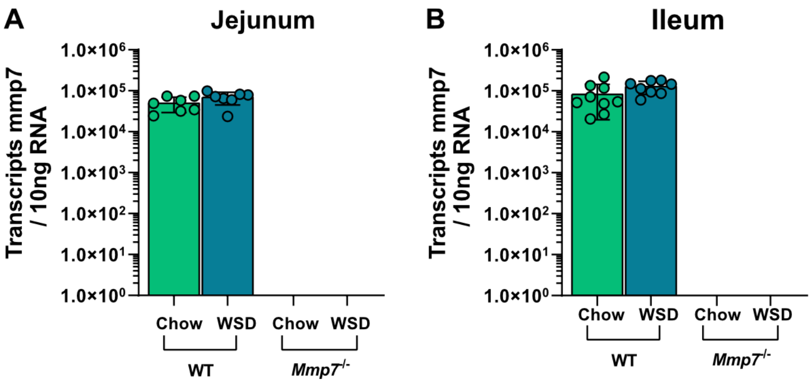

Supplementary Figure 1. Expression of *Mmp7*. *Mmp7* expression determined for WT and *Mmp7*$^{-/-}$ mice fed a chow or WSD at the jejunum (A) and at the ileum (B).

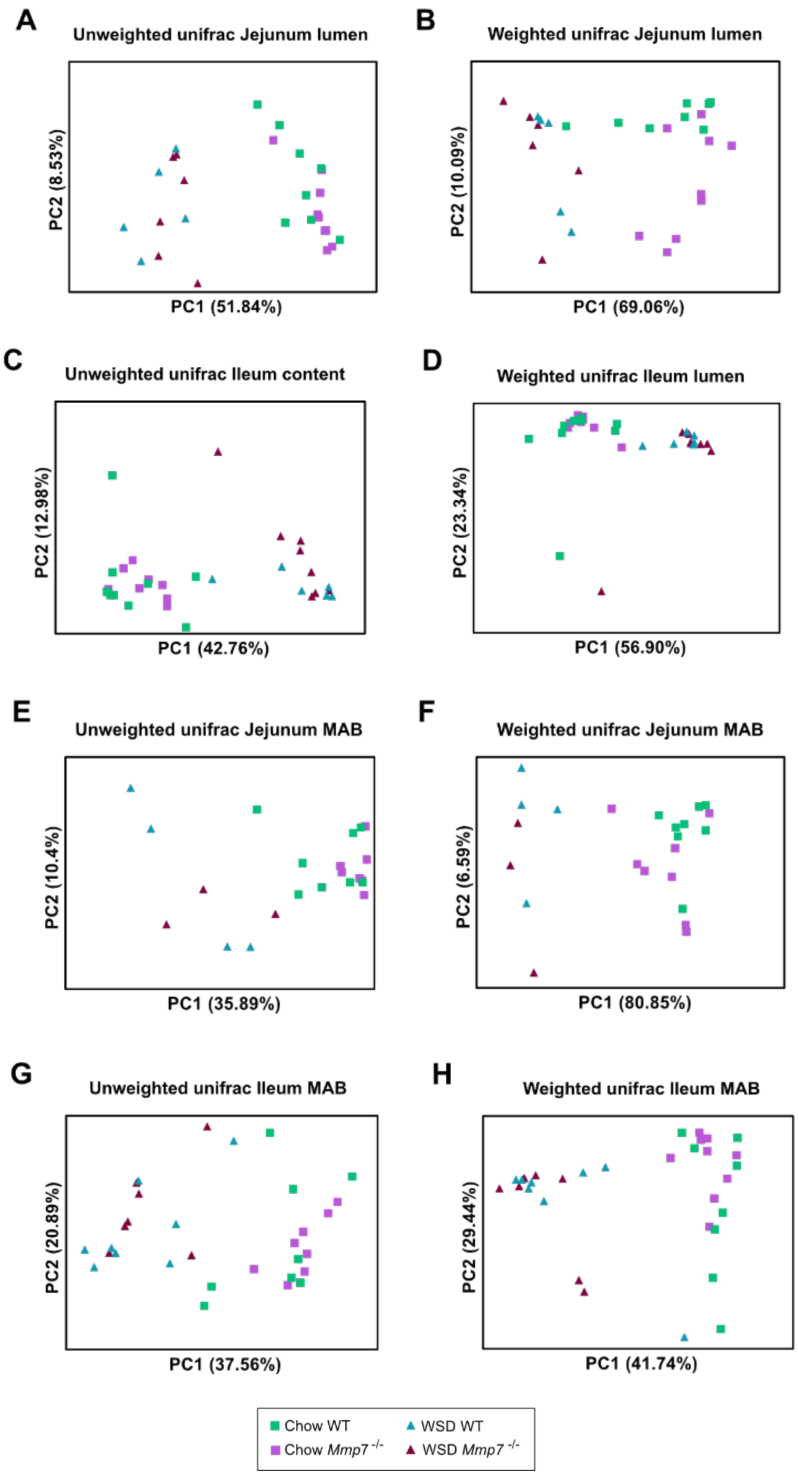

Supplementary Figure 2. β- diversity analysis of WT and *Mmp7*$^{-/-}$ mice fed a chow or WSD. Unweighted unifrac for jejunum content (A), ileum content (C), jejunum mucosa (MAB) (E) and ileum mucosa (G). Weighted Unifrac for for jejunum content (B), ileum content (D), jejunum mucosa (F) and ileum mucosa (H). Statistical significance was calculated with a PERMANOVA analysis ns= p> 0.05, *= p≤ 0.05, **= p≤ 0.01, ***= p≤ 0.001.

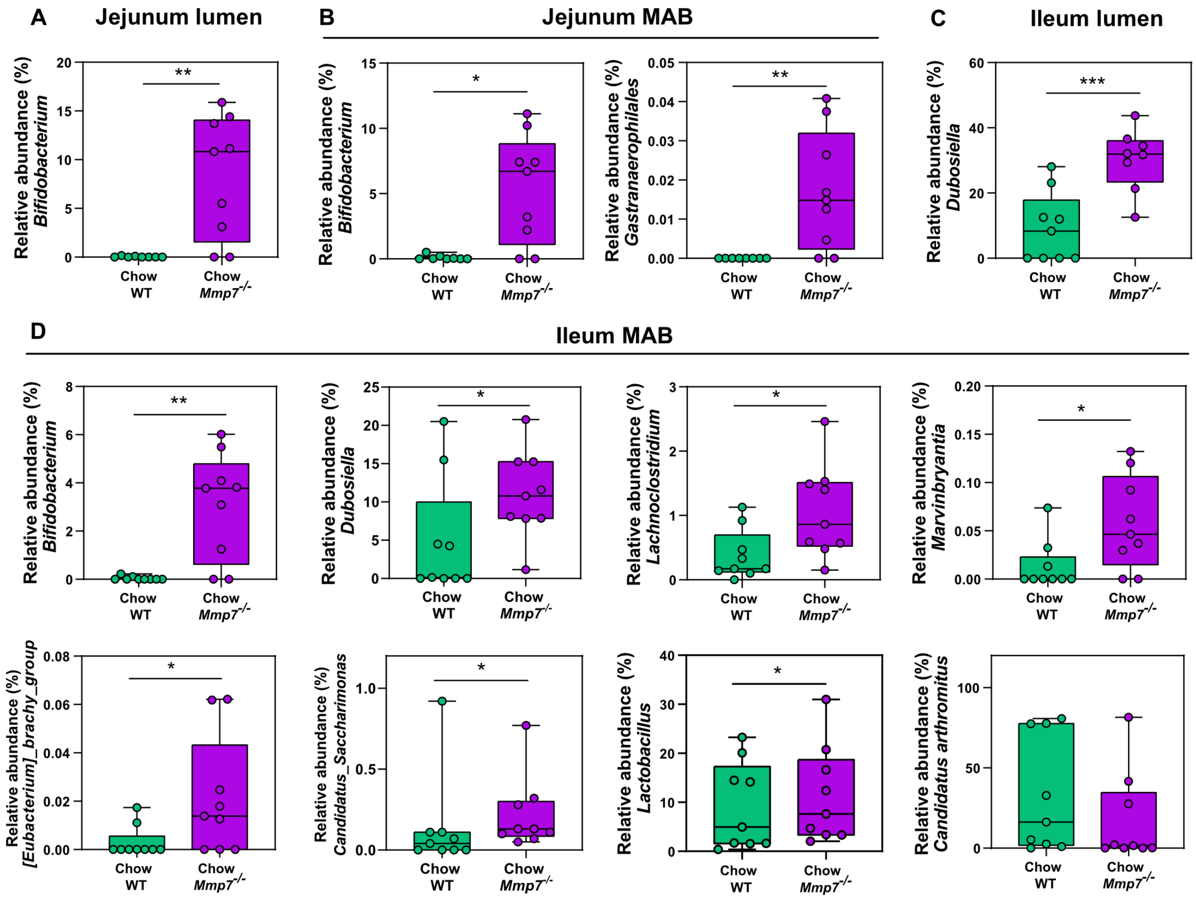

Supplementary Figure 3. Microbiota relative abundance. Relative abundance analysis of significantly enriched genera at the jejunum lumen (A), jejunum mucosa (MAB) (B), ileum lumen (C) and ileum mucosa (D) of chow-fed WT or *Mmp7*$^{-/-}$ mice. Statistical significance was calculated with a Mann-Whitney test ns= p> 0.05, *= p≤ 0.05, **= p≤ 0.01, ***= p≤ 0.001.

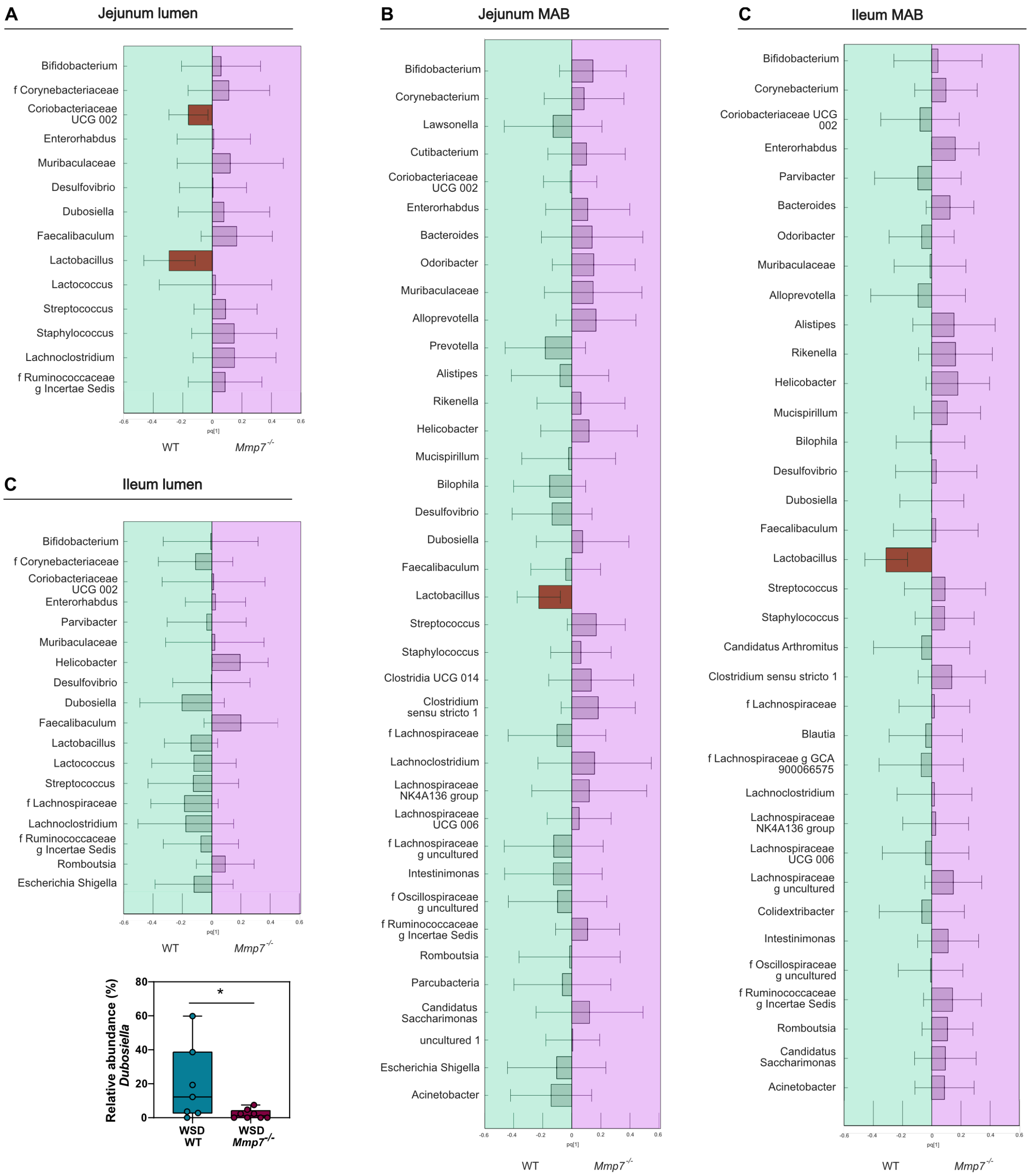

Supplementary Figure 4. OPLS-DA analysis between genotype in WSD fed mice. Orthogonal Partial Least Squares Discriminant Analysis (OPLS-DA) of bacterial genera in the jejunal lumen (A), at the jejunal mucosa (MAB) (B), in the ileal lumen (C) and at the ileal mucosa (D) of WSD-fed wild-type and *Mmp7*$^{-/-}$ *mice*. *Dubosiella* relative abundance in WSD fed mice in the ileal lumen (C). Statistically significant difference in bacteria relative abundance in the OPLS-DA analysis was defined when the confidence interval did not contain the null hypothesis value (0). For the relative abundance analysis, the statistical analysis was performed with a Mann-Whitney test ns= p> 0.05, *= p≤ 0.05, **= p≤ 0.01, ***= p≤ 0.001.

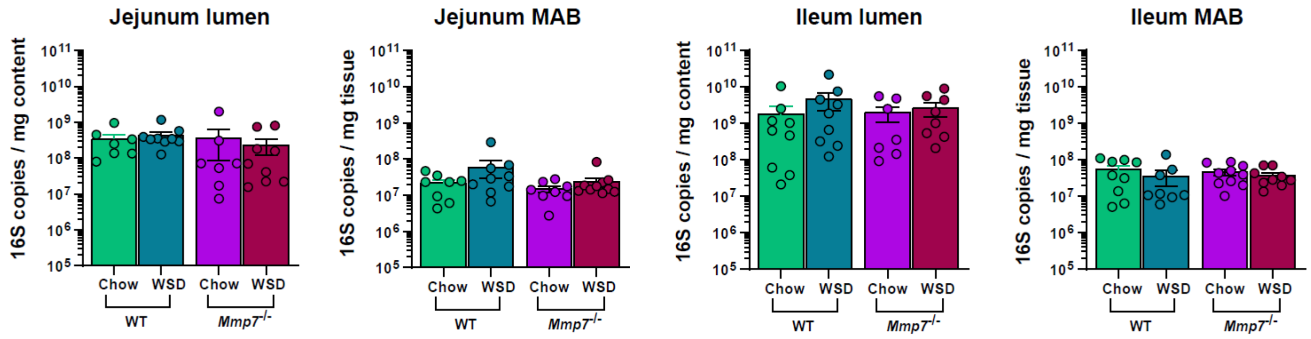

Supplementary Figure 5. Total bacteria quantification in chow- and WSD-fed WT and *Mmp7*$^{-/-}$ mice. The total bacteria amount was determined by measuring the 16S gene transcript copy number per mg of the jejunum and ileum luminal content, or per mg of tissue of jejunum or ileum mucosa (MAB). Statistical significance was determined by two-way ANOVA analysis with *= p≤ 0.05, **= p≤ 0.01, ***= p≤ 0.001.
